# Research evidence communication for policy-makers: a rapid scoping review on frameworks, guidance and tools, and barriers and facilitators

**DOI:** 10.1186/s12961-024-01169-9

**Published:** 2024-08-08

**Authors:** Jorge Otávio Maia Barreto, Roberta Crevelário de Melo, Letícia Aparecida Lopes Bezerra da Silva, Bruna Carolina de Araújo, Cintia de Freitas Oliveira, Tereza Setsuko Toma, Maritsa Carla de Bortoli, Peter Nichols Demaio, Tanja Kuchenmüller

**Affiliations:** 1 Oswaldo Cruz Foundation, Fiocruz, Brasília, Brazil; 2 Health Institute, São Paulo, Brazil; 3 McMaster Health Forum, Hamilton, Canada; 4 Department of Health, Aging and Society, McMaster University, Hamilton, Canada; 5 Evidence to Policy and Impact, Research for Health, Science Division, World Health Organization, Geneva, Switzerland

**Keywords:** Evidence-informed policy, Knowledge translation, Research communication, Policy-makers, Frameworks

## Abstract

**Background:**

Communication is a multifaceted process, ranging from linear, one-way approaches, such as transmitting a simple message, to continuous exchanges and feedback loops among stakeholders. In particular the COVID-19 pandemic underscored the critical need for timely, effective and credible evidence communication to increase awareness, levels of trust, and evidence uptake in policy and practice. However, whether to improve policy responses in crises or address more commonplace societal challenges, comprehensive guidance on evidence communication to decision-makers in health policies and systems remains limited. Our objective was to identify and systematize the global evidence on frameworks, guidance and tools supporting effective communication of research evidence to facilitate knowledge translation and evidence-informed policy-making processes, while also addressing barriers and facilitators.

**Methods:**

We conducted a rapid scoping review following the Joanna Briggs Manual. Literature searches were performed across eight indexed databases and two sources of grey literature, without language or time restrictions. The methodological quality of included studies was assessed, and a narrative-interpretative synthesis was applied to present the findings.

**Results:**

We identified 16 documents presenting either complete frameworks or framework components, including guidance and tools, aimed at supporting evidence communication for policy development. These frameworks outlined strategies, theoretical models, barriers and facilitators, as well as insights into policy-makers’ perspectives, communication needs, and preferences. Three primary evidence communication strategies, comprising eleven sub-strategies, emerged: “Health information packaging”, “Targeting and tailoring messages to the audience”, and “Combined communication strategies”. Based on the documented barriers and facilitators at micro, meso and macro levels, critical factors for successful communication of evidence to policy-makers were identified.

**Conclusions:**

Effective communication is indispensable for facilitating knowledge translation and evidence-informed policy-making. Nonetheless gaps persist in frameworks designed to enhance research communication to policy-makers, particularly regarding the effectiveness of multiple communication strategies. To advance in this field, the development of comprehensive frameworks incorporating implementation strategies is warranted. Additionally, barriers and facilitators to implementing effective communication must be recognized and addressed taking diverse contexts into consideration.

*Registration* https://zenodo.org/record/5578550

**Supplementary Information:**

The online version contains supplementary material available at 10.1186/s12961-024-01169-9.

## Background

The COVID-19 pandemic has shown that managing global health emergencies requires a robust evidence ecosystem. This ecosystem must not only produce evidence but also ensure its dissemination and use, enabling decision-makers at all levels of health systems to consider it in a timely manner, in their deliberations and in dialogue with society. The challenges of getting evidence in a timely, systematic and transparent way into decision-making processes for COVID-19 demonstrated failures in communicating results effectively and contextualizing the findings appropriately for local implementation contexts [1].

To address future challenges in using scientific evidence in government decision-making, enhancing dissemination and communication processes are essential.

In the context of health policy and systems, evidence-informed policy-making (EIPM) is a systematic and transparent process that integrates the research evidence on priority policy issues, into context-sensitive decision-making processes to drive change and achieve impact on health policy and systems [2]. EIPM is built on processes of translating evidence for practical application, known as Knowledge Translation (KT), which is defined as “the exchange, synthesis, and effective communication of reliable and relevant research results, with focus on promoting interaction among the producers and users of research, removing the barriers to research use, and tailoring information to different target audiences so that effective interventions are used more widely” [3]. Thus, effective KT requires different competencies, including those related to communication between researchers, policy-makers, and other stakeholders to address barriers to reach mutual understanding, and to improve research use and uptake [3].

EIPM requires policy-makers to have access to encompassing, relevant, and trustworthy evidence that is easy to understand and apply [4] and considers their communication needs, preferences [5], and political decision-making environment [6]. Barriers to reducing the research-to-policy gap include ineffective communication, and a lack of policy-maker skills to use research evidence [7, 8]. Additionally, policy-makers often rely on personal experience, selectively chosen evidence, public opinion, consultation feedback, or due to political and contextual pressures [6]. To improve the uptake of research in policy decision-making processes, evidence should be structured and packaged in a way that is actionable and relevant for policy-makers, including information on competing interests and other sources of information [9]. Consequently, effective communication between researchers and policy-makers may require more complex and long-term strategies to set up links and promote exchanges between these groups, as well as specific frameworks and tools to help guide the communication of evidence.

Major EIPM initiatives building on lessons learned from COVID-19 such as The Global Commission on Evidence [10] and the World Health Organization’s (WHO) “Together on the road to evidence-informed decision-making for health in the post-pandemic era: a call for action” [11], have recently stressed the importance of effective communication in promoting the uptake of research evidence in decision-making. For example, the Global Commission on Evidence report recommends that evidence groups prepare “derivative products” to communicate evidence tailored to their target audiences, including informational needs, and adopt formats that help understanding of key messages and delving deeper if interested [10]. Likewise, WHO’s call for action included recommendations for governments and intergovernmental organizations to build national and international capacity to translate, communicate, and support the use of evidence in decision-making [11].

This scoping review aimed to identify and classify the global evidence on frameworks, guidance and structured tools that support the communication of research evidence to policy-makers. The review focused on better addressing their needs, preferences, perspectives, as well as overcoming barriers and leveraging facilitators in research communication.

## Methods

### Study design and protocol

We conducted a rapid scoping review [13] according to the Joanna Briggs Institute Reviewer’s Manual [14] and the PRISMA Extension for Scoping Reviews [15]. A prospective protocol is available [16].

### Research questions and eligibility criteria

PCC acronym: Population (P): policymakers; Concept (C): Preferences, perceptions, needs, guidance, tools and frameworks to support effective communication of research evidence; Context (C): Evidence-informed policy-making (EIPM); were used to define two research questions: (1) What are the available frameworks, including guidance and structured tools, to support the evidence communication to policy-makers? and (2) What are the barriers and facilitators, including policy-makers' perspectives, needs, and preferences, regarding evidence communication?

### Population

Given the various definitions of policy-makers exist, we included studies identified as having the authority to make decisions about health policies and programs at any level. This includes both individual decision-makers and collective bodies such as councils and participatory structures. We excluded studies involving other stakeholders, such as health professionals, civil society members, or health services users. We also excluded studies with participants whose decision-making power was limited to medical or clinical matters.

### Concept

We selected documents that presented frameworks, guidance, and structured tools to support effective communication of research evidence to policy-makers. This included those that were part of a larger framework, such as a broader knowledge translation framework. For this study, we used a broad concept of ‘framework’, defining it as any systematization of theoretical or practical elements based on existing theories, including assumptions and any other components of theoretical foundation and practical application. Our focus was on how these structures incorporated policy-makers' perspectives, needs and preferences, while identifying the barriers or facilitators to these communication processes.

Considering the objective of this review, authors defined "framework” as any systematization of elements to support the communication of evidence to decision-makers. Thus, frameworks can be comprehensive or focus on a specific part of the communication process.

Furthermore, for the purpose of this review, we defined “guidance” as any structured set of recommendations on how to carry out evidence communication, including the identification of barriers / facilitators, as well as strategies for addressing these barriers. We defined “structured tool” as any mechanism, process, or support resource designed to aid in communicating evidence.

### Context

For this scoping review, we recognize that the communication of evidence is a complex, multifaceted process, influenced by contextual factors. Therefore, we included frameworks, guidance, or structured tools applied to health policy and systems decision-making contexts across various levels of jurisdiction, ranging from local to global, and organization levels, spanning from micro, meso to macro level.

### Study designs

The review included primary and secondary research, qualitative and quantitative approaches).

### Settings

We imposed no restrictions on the country from which data was collected or on the level at which decisions were made. We included studies referencing other social policy sectors, provided they also addressed a health policy or health systems issue. However, studies solely focused on sectors such as education or economics, were excluded if no clear connection to health policy was evident. Additionally, we excluded documents that lacked clear descriptions of the policy-making context.

### Time and language

There were no restrictions on the date of publication or language.

### Information sources and search strategies

The searches were conducted on 23 October 2021, in eight electronic databases: PubMed, Virtual Health Library Regional Portal, Embase, Cochrane Library, Health Systems Evidence, Social Systems Evidence, Epistemonikos, and Scopus. Grey literature was searched on Opengrey and Google Scholar. The search strategies were adapted for each database. Appendix 1 provides the search strategies in detail. In addition to the searches, EIPM experts, external to the research group, were consulted, and they suggested additional documents of relevance to this review.

### Selection

Two pairs of reviewers (BCA, CFO, LALBS and RCM) independently screened titles and abstracts, using Rayyan [17]. Disagreements were resolved by consensus, and when needed, a third reviewer (MCB or TST) was consulted. Full-text documents were assessed by one reviewer and checked by another (BCA, CFO, LALBS and RCM).

### Data extraction and categorization

We used a data extraction form developed by our team to extract the characteristics of the studies and relevant information according to predefined categories outlined in the protocol. One reviewer conducted the data extraction, with validation performed by another member of the team (BCA, CFO, LALBS or RCM). Instances where data were unavailable were documented as “not reported”. The characteristics and categories extracted included: study identification (lead author, year of publication, study design); aim/s or objective/s; studies’ focus; study participants and settings (countries where the research was undertaken, types of participants, healthcare setting/s in which the study was carried out); results/findings (message presentation, communication channels, evidence communication framework, guidance or tools, policy-makers perspectives, needs and preferences, conclusion, limitations, and research gaps). Regarding the policy-makers’ perspectives, needs, and preferences about research communication, the data were analyzed and coded into similarity categories (barriers, facilitators, and future actions).

We used the categories outlined by Blessing et al. [18] to organize our findings based on the information gathered during data extraction. These categories encompass evidence communication strategies aimed at presenting information in a user-friendly format, tailored to diverse audiences, incorporating various means of delivering the information. Blessing et al. identified four broad categories for these strategies: packaging tools, application tools, dissemination and communication tools, and linkage and exchange tools. Additionally, we coded information about the format and means of communication according to these categories, accounting for diverse mechanisms or communication modalities aimed facilitating the assimilation of health information by policy makers.

### Methodological appraisal of the included studies

The quality of the studies was assessed using The Joanna Briggs Institute critical appraisal tools (https://jbi.global/critical-appraisal-tools), considering the methodological design of each study.

## Results

### Literature search

**Fig. 1:**
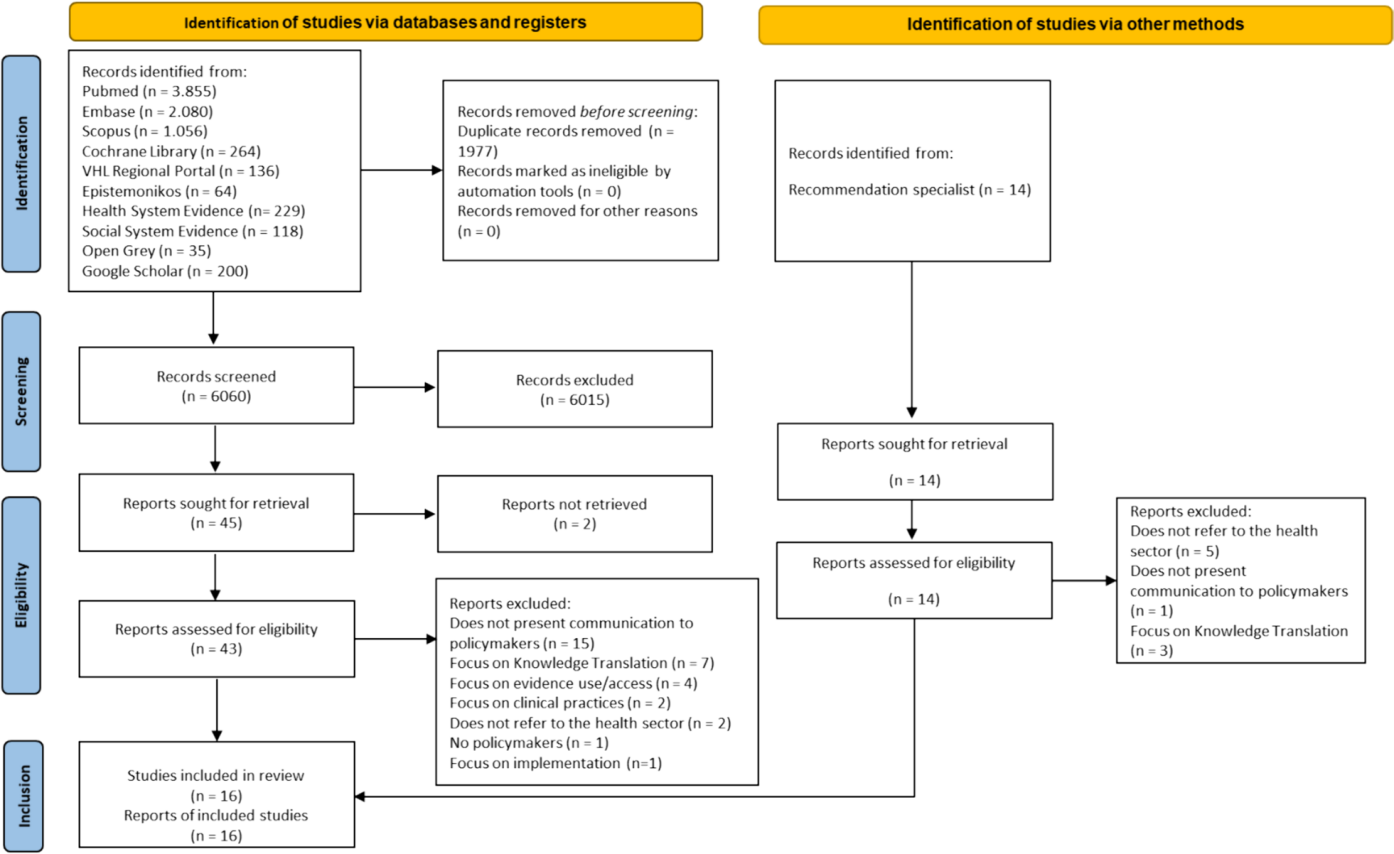
Study selection flow diagram, adapted from PRISMA 2020[31]

Our searches identified 8037 records. After 1977 duplicates were removed, we screened 6060 records, of which 6015 were excluded because they did not meet the eligibility criteria. The experts consulted suggested fourteen additional documents for consideration. Only 16 documents met all the eligibility criteria (primary and secondary qualitative or quantitative studies; policymakers preferences, perceptions or needs; guidance, tools and frameworks to support effective communication for evidence-informed policy-making) and were included in this scoping review [5, 8, 12, 18–30] (Fig. 1). The list of excluded studies with the reasons for exclusion is provided in Appendix 2.

### Characteristics of the included studies

The included studies were published from 2002 to 2021. Study designs included: systematic reviews [8, 19, 21, 27, 29]; a scoping review [18]; an overview of systematic reviews [12]; a qualitative review combined with a survey [28]; a qualitative study [22]; a quality assurance exercise [30]; a fundamental qualitative descriptive design [5]; a qualitative exploratory study [24]; a mixed qualitative methods case study [26]; and a technical report that includes a systematic review of reviews (overview) and a scoping review [20]. Two studies did not inform their own methodological designs, but for the purpose of evaluating the methodological quality, we categorized them as one non-systematic literature review [23] and one qualitative study [25].

The primary studies, including those reported in the selected reviews, were carried out in: low- and middle-income countries (LMIC) [20], high-income countries [20, 21, 25–27, 29, 30], and a mix of both LMIC and high-income countries [12]. Six publications did not provide any information on the countries in which the primary studies were conducted [5, 8, 18, 19, 22, 28].

Among the studies included in this scoping review, thirteen of them described the roles and responsibilities of individuals identified as policy-makers [5, 12, 18, 20–27, 29, 30], seven of which presented a specific definition [12, 19–22, 26, 27]. Thirteen studies were conducted in healthcare settings [5, 8, 12, 19–21, 23–28, 30], six of which also involved other sectors [8, 20, 22–24, 28]. Two studies did not specify the settings [18, 29], but health messages were communicated to policy-makers.

Additional characteristics of the included studies are presented in Table 1 and detailed in Appendix 3.

**Table 1:** Characteristics of the included studies

Lead author	*Study design	Number of included studies	Countries focused on the research question (number of studies)	Study participants or settings	Type of finding	Conflict of interest
Ashcraft et al. (2020) [29]	Systematic review	27 primary studies	USA (*n* = 10)	Policy-makers (general); State-level policy-makers; State mental health executive; County Health Department officials; State legislative policy-makers; Members of public policy agencies, national organizations, and congressional staffers; State Medicaid Directors and Pharmacy Directors	Tools	The authors declare no conflict of interest
Blessing et al. (2017) [18]	Systematic review	54 primary studies	Not reported	Stakeholders: Experts/researchers, policy-makers, health practitioners, civil society, NGOs, media, citizens	Tools Frameworks	Not reported
Campbell et al. (2009) [30]	Quality assurance exercise (interview with researchers and policy-makers)	Not applicable	Australia	Policy-makers (general): employed at senior levels of the NSW Department of Health, Area Health Services	Tools	The authors declare no conflict of interest
Chapman et al. (2021) [12]	Overview	27 systematic reviews	Australia (*n* = 18), Bangladesh (*n* = 3), Brazil (*n* = 1), Burkina Faso (*n* = 4), Cambodia (*n* = 1), Cameroon (*n* = 1), Canada (*n* = 57), China (*n* = 4), England (*n* = 8), Ethiopia (*n* = 1), Fiji (*n* = 2), France (*n* = 1), Germany (*n* = 1), Ghana (*n* = 1), Guatemala (*n* = 1), Hungary (*n* = 1), Iran (*n* = 2), Kenya (*n* = 3), Mexico (*n* = 3), Nepal, (*n* = 1), Netherlands (*n* = 7), Nigeria (*n* = 3), Norway (*n* = 1), Philippines, (*n* = 1), Republic of Ireland (*n* = 1), Rwanda (*n* = 1), Scotland (*n* = 1), Spain (*n* = 1), South Africa (*n* = 6), Switzerland (*n* = 1), Taiwan (*n* = 1), Tanzania (*n* = 1), Uganda (*n* = 2), USA (*n* = 22), UK (*n* = 25), Vietnam (*n* = 2), Zambia (*n* = 3)	Decision-makers: hospital directors or administrators, health administrators, department chiefs, health planners, and programme directors or managers	Tools	The authors declare no conflict of interest
Dobbins et al. (2004) [25]	Not reported (qualitative study with analysis of 9 focus groups)	Not applicable	Canada	Medical officers of health, public health managers and directors, health promotion managers, and health policy-makers at provincial and federal levels	Tools	Not reported
Dobbins et al. (2007) [5]	Fundamental qualitative descriptive design	Not applicable	Canada	Among the participating health units, purposive sampling was used to find decision-makers who, in their roles, make decisions related to public health practice and policies, in other words, participants managed decisions concerning provision of services, rather than directly supplying services to the public. Respondents were programme managers (*n* = 9), programme directors (*n* = 6), and a Medical Officer of Health (*n* = 1)	Tools	Not reported
Funk et al. (2021) [19]	Scoping review	17 studies	Not reported	Policy-making is a political, complex and dynamic process, with multiple actors involved having different interests, roles, and resources	Tools Frameworks	The authors declare no conflict of interest
Innvaer et al. (2002) [27]	Systematic review	24 studies	USA (*n* = 10), USA and UK (*n* = 1), UK (*n* = 3), Canada (*n* = 3), Australia (*n* = 1), Burkina Faso (*n* = 1), Mexico (*n* = 1), Netherlands (*n* = 1), Pakistan (*n* = 1), South Africa (*n* = 1), Sweden (*n* = 1)	We only included studies of health policy-makers responsible for decisions on behalf of a large organization or jurisdiction, people in ‘upper-level positions’ making important policy decisions, such as "senior managers", and staff members responsible for decisions	Guidance (Barriers and Facilitators)	Not reported
Langer et al. (2016) [20]	1. systematic review of reviews (overview) 2. scoping review**	Overview (36 systematic reviews) Scoping review (67 studies)	High-Income Countries (*n* = 9), Low- and Middle-Income Countries (*n* = 2)	Dep. of Health programme directors, Clinical supervisors, Social workers and policy-makers, Public health policy-makers, Nurses	Tools Frameworks	Not reported
Lavis et al. (2003) [28]	Qualitative review and survey	Not reported	Canada	Target audience—1) public/service recipients (e.g., citizens, patients and clients), 2) service providers (e.g., clinicians), 3) managerial decision-makers (e.g., managers in hospitals, community organizations, and private businesses), and 4) policy decision-makers at the federal, state/provincial, and local levels	Tools	This study was funded through an open grants competition by the Ontario Ministry of Health and Long-Term Care
McCormack et al. (2013) [21]	Systematic review	61 articles (representing 54 studies)	USA, Hong Kong, Canada, England, Germany, Finland, Netherlands, Scotland, Spain, and Switzerland	Not reported	Tools Framework	The authors declare no conflict of interest
Meisel et al. (2019) [22]	Qualitative study	Not applicable	USA	Decision-makers from health care delivery systems, the insurance industry, the pharmaceutical industry, clinical care settings, federal and state governments, and patient advocacy organizations. Policy-makers were broadly defined as leaders and decision-makers from a variety of sectors who might use research to influence healthcare related to substance use disorder	Guidance (Barriers and Facilitators)	The authors declare no conflict of interest
Oliver et al. (2014) [8]	Systematic review	145 studies	Not reported	The population samples were predominantly policy-makers or advisors, health care managers, researchers, physicians, local authority staff, allied health professionals, information/surveillance staff, surgeons, legal staff, midwives. Other participants included commissioners, health economists, third sector workers, patients, industry and business representatives, and justice and criminal workers	Tools	The authors declare no conflict of interest
Purtle et al. (2020) [23]	Not reported (literature review)	Not reported	USA	State legislators, directors of state mental health agency, and senior staff	Tools	The authors report no financial relationships with commercial interests
Schmidt et al. (2014) [24]	Qualitative exploratory study	Not applicable	USA	Former state senators and representatives	Tools	The authors declare no conflict of interest
Wye et al. (2015) [26]	Mixed qualitative methods case study	Not applicable	Canada	Healthcare commissioners; Those charged with delivering ‘evidence-based policy-making’ within the English National Health Service	Tools	Not reported

*Study type: When the authors did not inform the study design, the reviewers attributed a classification based on the description of the methods provided. **This publication is part of the project “Science of Using Science”, and its final product is a report with two types of research. Abbreviations: CEOs—chief executive officers; HPAC—Health Policy Advisory Committee; KB—Knowledge Brokers; LMIC—low- and middle-income countries; MoHP—Ministry of Health and Population; NGOs—non-governmental organizations, NSW—New South Wales; PFP—private-for profit sector; PNFP—private not-for-profit; UK—United Kingdom; USA—United States of America

### Methodological assessment of included studies

We assessed the methodological quality of the included studies. Five systematic reviews [8, 18, 21, 27, 29], one overview of systematic reviews [12], one scoping review [19], and one report that combined an overview and systematic review of reviews (overview) with a scoping review [20] were evaluated with the JBI Checklist for Systematic Reviews, but they did not provide information on quality assessment of primary studies or on strategies to minimize data extraction errors. Five of seven studies evaluated with the JBI Checklist for Qualitative Research did not report on influence of researchers in conducting the research [5, 24, 26, 28, 30]. One study [23] evaluated with the scale for the quality assessment of narrative review articles (SANRA) had a score of 8/12, due to the lack of a detailed description of the searches, and the lack of presentation of the designs of the included studies [23]. Details of the methodological quality assessment are provided in the Appendixes 4.1 to 4.3.

### Categorization of findings on evidence communication strategies

We found four studies that presented comprehensive frameworks to guide the evidence communication to policy-makers [18–21].

From the perspective of Blessing et al. [18] and Funk et al. [19], relevant mechanisms for knowledge translation require structured communication processes, including:

- push efforts—providing knowledge to users in appropriate formats;
- push methods—information producers use research data and health information to create various products;
- pull efforts—enabling policy-makers to identify relevant information;
- pull methods—policy-makers commissioning a summary of evidence based on exact specifications;
- pull efforts by end-users—e.g., through knowledge brokering;
- linkage and exchange efforts—to build relationships between producers and users of health information;
- integrated methods—a knowledge translation platform institutionalized in an organization or in the health system [18, 19].

To analyze the findings of studies that did not include comprehensive frameworks, but communication strategies or tools that were part of other integrated frameworks, we used a combination of the categories proposed by Blessing et al. [18], Funk et al. [19], Langer et al. [20] and McCormack [21], covering a wider range of categories that may overlap, but with slightly different nomenclatures. Thus, the findings of this section are categorized into an eclectic framework that aggregates categories from these four studies (Table 2).

**Table 2:** Categories of communication strategies or tools

Tools [18, 19]	Techniques [20]	Strategies [21]
Packaging tools Application tools Dissemination and communication tools Linkage and exchange tools Linking tools to intended outcomes	Tailoring and targeting Framing Online and social media Branding Reminders Timing Information design Audience segmentation Access options	Tailoring the message Targeting the message Using narratives Framing the message. Improve reach of evidence Motivate recipients to use and apply evidence Enhance recipients’ ability to use and apply evidence More than one strategy

It is important to note that Langer et al. [20] and McCormack et al. [21] presented “communication” and “dissemination” strategies separately, however for the purpose of this review, we considered these strategies together as communication processes involving evidence and decision-makers. The strategies for evidence communication are presented in Appendix 5.

We categorized communication strategies or tools into four groups: Health information packaging tools; Targeting and tailoring the messages; Strategies to improve reach of evidence; and Combined communication strategies (Table 3).

**Table 3:** Summary of results on communication strategies

Strategies	Categorization of findings
Health information packaging	
Evidence synthesis	• Brief summaries, full summaries [28]; brief research summaries, summaries of local data [30]; summaries, executive summary [5] research brief [23]; evidence summaries [12, 23]; summary of research [29]; summary statement [25]; meeting papers [26] • Policy briefs [18, 23, 24, 28, 29] or evidence-briefs for policy [19] • Brochures, fact sheets, report cards, press releases [29]; local health memoranda (or messages) [18]; memos to/from the government [19]
Visualization or information design	• Arts-based projects [12] • Graphs and charts (line graphs, bar charts or pie charts), infographics, data dashboards, dynamic graphs [18] • Publicly-accessible visualization platform (included geographical maps) [19]; maps and e-atlases [18]; maps using geographic information systems, policy map (a web-based data mapping tool) [23] • Entertainment education—prime-time network TV storyline, short films [12]
Narratives	• Narratives, booklets with testimonies, advocacy summaries of individual experiences [12] • Strategic frames (for example, brief narratives or stories) [23]; Narratives (enhancing existing evidence communication practices to increase the relevance and accessibility of research results) [20]
Targeting and tailoring the messages to the audience	
Targeting the message	• Fitting evidence-informed decision-making promotion / research message to decision-maker audience [20] • County health rankings report [29]; Public health reports [18]; National and regional directives, performance, activity, financial and referral data, business cases [26] • Guidelines, pathways [26] • Systematic reviews [5, 12, 20, 30]; Original legal research article [29]; Literature reviews and summary reports [29] • Communication designed for an individual based on information from the individual [21]
Tailoring the message	• Tailoring strategies (content matching, personalization, and feedback) [21] • Individualized feedback (e.g., via chat, telephone, or face to face) [21]
Improve reach of evidence	
Electronic tools for dissemination and communication	• Platforms for sharing health information [12]; national clinical databases [18]; platforms where substantial amounts of data are stored with open-access, interactive data platforms, or surveillance platforms [19] • Repository of systematic reviews [12]; Cochrane; database access [12]; Online evidence portals [20]; Online database of systematic reviews + weekly targeted messages [20]; Evidence portal and systematic review summaries + dissemination of evidence exclusively to decision-makers who had initially expressed an interest in it [20] • Online repositories, engagement prior to providing the opportunity, as well as offering multiple means of access [20] • Websites [12, 28]; web-based information and communication, dissemination of systematic reviews through the website [12] • Relevant research evidence reports distributed through public health professional organizations or through a clearinghouse [5] • Online/electronic service, hard copy [25]; electronic copy [25]; Electronic and hard copy print materials [29] • Print or Internet [21]
Tools for automated electronic dissemination of information	• Letters and memos [19]; targeted messaging [12] • Newsletters or e-mail containing summaries of current research [5] • Newsletters [18, 28] • Media outreach campaign via websites, e-mail listserv [29]; e-mail messages [18]; tweets or phone messages [18]\ • Series of e-mails with links to full references, abstracts, and summaries [12]; e-mails [28] • Reminders, incentives, framing and anchoring [20]
Online and social media	• Social media [29] • Wikis, blogs [12] • Applying online & social media tools to increase the reach and convenience of evidence and communication to strengthen evidence-informed decision-making [20]
Mass media	• Media and public opinion, television appearances, entertainment education—prime-time network TV storyline, short films, wikis, blogs, and online forums [12] • Radio spots [8] • Public education campaign [29]
Person-to-person communication	• Online forums [12] • Informal approaches from researchers [30]; one-to-one interaction with the researcher to discuss research findings [5]; one-way (and sometimes one-off) processes (i.e., beyond producer-push efforts) [28] • Conferences [18, 29, 30]; conference technology [12]; webinars [29]; forums, health forum [18, 30]; teleconferencing and webinar facilities [18]; conference technology to support knowledge sharing [12]; web/tele/face to face conferencing or some type of mixed mode session, recorded web-conferencing, Elluminate Live V-Class (web-conferencing tool), meetings [28]; seminars [23]; structured seminar series [12] • Person-to-person communication [29]; face-to-face interactions [12] • Regular public stakeholder meetings, oral presentations, discussions, deliberative dialogues [18]; chance encounters, formal meetings, and informal gatherings [26]; oral presentations, where one or several persons can present health information to a specific audience, such as policy-makers [19]; deliberative dialogue [12, 19]
Linkage and exchange tools	• Communities of practice, networks [12]; Stakeholder networks [18]; and interpersonal connections with staff, tailored exchanges within and across departments and disciplines [12] • Knowledge networks [12, 18]; platforms [12, 18]; evidence champions or brokers [18, 29]; individual knowledge brokers [12, 18, 28]; opinion leaders [28] authoritative endorsement by a respected organization [28] • Interventions designed to motivate target audiences to use and apply evidence (e.g., knowledge brokering) [12, 20]; evidence champions or brokers [18, 29]; individual knowledge brokers [12, 18, 28]; opinion leaders [28] authoritative endorsement by a respected organization [28] • Capability, motivation, and opportunity (CMO): targeted communication, audience segmentation in dissemination, and more accessible and user-friendly packaging of evidence [20]
Enhance recipients’ ability to use and apply evidence	• Web-based training programs, structured seminar series [12] • Interventions to enhance recipients’ ability to use and apply evidence (e.g., training workshops with an interactive component) [12]; workshops [18]; small-group meetings [29]; small-group workshops, narrative action reflection workshops [12]; exchange and integrated knowledge translation mechanisms [19]; organization-wide capacity-development initiatives [12] • Interventions designed to motivate target audiences to use and apply evidence (e.g., knowledge brokering) [12, 20]
Combined communication strategies	
More than one of the above strategies	• Combined communication strategies, tailored and targeted messages (both strategies are usually implemented together) [12] • Interventions that incorporate two or more distinct strategies (i.e., that are multifaceted) are consistently more likely to work than single interventions [21] • Online database of systematic reviews, weekly targeted messages, and KB (knowledge broker) that did not find a positive effect of applying M3 (communication & access) [20] • Motivation to use evidence was created through a more personalized and targeted manner of evidence communication. The combination of building motivation and opportunity to use evidence succeeded in encouraging decision-makers’ use of evidence as measured by the number of actual evidence-based strategies, policies, and interventions being implemented as well as the reported use of systematic reviews to inform a policy decision in a two-year period [20]

The strategies “Health information packaging tools” are composed of synthesis tools [5, 12, 18, 19, 23–26, 28–30], visualization tools [12, 18, 19, 23], and narratives [12, 20, 23]. The strategies “Targeting and tailoring the messages to the audience” refers to Targeting the message [5, 12, 18, 20, 21, 26, 29, 30] and Tailoring the message [21]. The “Strategies to improve reach of evidence” include: electronic tools for communication [5, 12, 18–21, 25, 28, 29], automated electronic dissemination of information [5, 12, 18–20, 28], online and social media [12, 20, 29], mass media [8, 12, 29], person-to-person communication [5, 12, 18, 19, 23, 26, 28–30], linkage and exchange tools [12, 18, 20, 28, 29], enhance recipients' ability to use and apply evidence (regardless of delivery mode) [12, 18–20, 29], and combined strategies [12, 20, 21].

Most strategies were reported in only one study; however, the findings related to “Targeting the message” as brief forms for the presentation of evidence were the most cited, with different nomenclatures, such as: evidence summaries (*n* = 7) and policy briefs (*n* = 6). In addition, there was a higher frequency of findings of synthesis tools, a category composed of systematic reviews (*n* = 4) and public health program reports (*n* = 3).

Among the findings about the “Strategies to improve reach of evidence”, the most frequently found were Conferences (*n* = 4), Newsletters (*n* = 2), E-mails (*n* = 2), Websites (*n* = 2), and Deliberative dialogues (*n* = 2). In addition, Information linkage and exchange tools were reported in five studies [12, 18, 20, 28, 29], including focusing on individual knowledge brokers (*n* = 3), evidence champions or brokers (*n* = 2), and networks (*n* = 2). Enhance recipients' ability to use and apply evidence (regardless of delivery mode) was most frequently observed in two categories: workshops (*n* = 2) and Interventions designed to motivate target audiences to use and apply evidence (e.g., knowledge brokering) (*n* = 2).

Combined communication strategies refer to messages that can be implemented together and at the same time [12, 20, 21].

### Barriers and facilitators on evidence communication to policy-makers

Perceptions of policy-makers were reported in 13 studies [8, 12, 20–30], aggregated into factors that can hinder (barriers) or facilitate (facilitators) the communication of research evidence.

Barriers were divided into eight thematic categories, applicable to the three levels of an organization (micro, meso and macro level) according to RNAO [32]. The lack of access to information (*n* = 8), as well as of the relevance of the information (*n* = 8) were mentioned the most frequently. Facilitators were also divided into eight thematic categories, highlighting the format/content of the materials (*n* = 7), and relationship between researchers and policy-makers (*n* = 5). The small numbers of studies could be misleading in terms of frequency counter. Thus, our analysis of these elements considers the frequency with which they appeared across studies. The Table 4 presents the barriers and facilitators found for each of the influencing factors identified at the micro-, meso-, and macro-levels. More details are available in Appendix 6.

**Table 4:** Number of studies that presented findings for barriers and facilitators in each organization level

Organization Levels	Barriers	Facilitators
Macro-level		
Political or organizational Instability	3	0
Meso-level		
Institutionalization/culture for the use of scientific evidence	3	0
Material and human resources	3	0
Knowledge brokers participation	0	1
Diversity in communication and dissemination channels	0	2
Encouraging the production and use of evidence and knowledge translation plans	0	3
Micro-level		
Customized and specific products	0	5
Ability to effectively use evidence	3	0
Relationship between researchers and policy-makers	3	5
Format and content of materials	7	7
Relevance of the information	8	3
Access to information	8	4

Many influencing factors (barriers and facilitators) reported in the studies appear at the micro level, which corresponds to the individual level, addressing policy-makers’ attitudes, beliefs, and knowledge. At this level, the following was pointed out as barriers: (1) the format for presenting evidence [8, 12, 22, 23, 25, 26, 30], for example excessive information or inadequate language; (2) difficulty in accessing evidence [8, 12, 22–24, 26, 27, 30], which may be due to an untimely delivery; (3) lack of relevant or good quality research [8, 12, 22, 26–30] that often does not respond to the decision-maker’s needs; (4) lack of ability to deal with evidence, mainly due to lack of training to understand the data [8, 12, 26]; and (5) low collaboration between researchers and policy-makers in the research partnership or alignment between interests [12, 27, 30].

Still at the micro level, facilitators included: (1) format and content of materials [8, 12, 20, 21, 23, 25, 27], since the message needs to be simple, well-written, clear, concise, easy to read, transparent, and well-organized; (2) relevance of the information produced for policy-makers [23, 25, 27], especially the credibility of research and its applicability to the local context; (3) personalized and specific products for the target audience [8, 20, 23, 25, 29], such as the option of printed or online materials; easy access to information [8, 20, 25, 27] with availability of electronic repositories for the materials produced, and guarantee of internet access for dissemination of content; and (4) relationship between researchers and policy-makers [8, 22, 27, 29, 30], which allows, for example, a pre-established dialogue and increased trust between the parties, and the understanding of policy-makers about research results.

The meso level includes aspects of leadership, culture, and available resources within the organization. At this level, the following was identified as barriers: (1) the lack of institutionalization/culture for the use of scientific evidence [12, 29, 30], since policy-makers are not sensitized to use evidence or are unaware of the need to use them in decision-making; and (2) the lack of material/human resources [12, 25, 27], especially internet access, printed materials, as well as specialists to carry out searches for evidence.

The identified facilitators at meso level were: (1) diversity of communication channels [23, 24], which can have multiple approaches and be customized to sensitize policy-makers; (2) participation of knowledge brokers [21] (professionals capable of bringing quality, relevant and effective information to policy-makers); (3) encouraging the production of evidence and knowledge translation plans [8, 20, 27] to assist the communication process; and (4) engagement of policy-makers in research, and in conducting studies and prioritizing the themes to be investigated, based on the needs of health systems.

The macro level encompasses the system, where the extent to which aspects of change are aligned with existing policies and government standards is verified. The barrier identified at this level was political or organizational instability that can be seen in situations such as turnover of managers, sectoral reform processes, or political interests [27]. No facilitators were reported by the studies.

### Future perspectives on evidence communication to policy-makers

In order to systematize the strategies to improve evidence communication to policy-makers, we organized our findings into five categories, linked to the influencing factors presented in the earlier section. The categories and number of studies that referred to each set of strategies were: Format/Content of materials/Delivery mode (*n* = 6); Relationship between researchers and policy-makers (*n* = 4); Relevance of information (*n* = 3); Institutionalization/Culture for the use of scientific evidence (*n* = 2); and Access to evidence (*n* = 1).

Similarly, information on strategies to improve evidence communication to policy-makers was more frequent at the micro level. The included studies did not report macro-level perspectives (Table 5). Details of each category are available in Appendix 7.

**Table 5:** Number of studies that presented findings on future perspectives

Organization levels	Studies
Meso-level	
Institutionalization/Culture for the use of scientific evidence	2
Micro-level	
Access to evidence	1
Information relevance	3
Relationship between researchers and policy-makers	4
Format/Content of Materials/ Mode of Delivery	6

Strategies to improve evidence communication to policy-makers at micro level included: (1) format and content of materials and the delivery mode [5, 22, 23, 25, 28, 30]; (2) useful recommendations for the presentation of evidence; relevant information; (3) delivery time for the response to the decision-maker; (4) the need for executive summaries of evidence; relationship between researchers and policy-makers [22, 26, 29, 30]; (5) involving researchers in policy discussions; fostering mutual trust; (6) more efficient formats of interpersonal contact; and (7) relevant evidence [5, 25, 26], demand-driven and needs-based needs as well as actionable evidence [25] with recommendations for making evidence available in a timely manner.

At the meso level, the identified strategies included: (1) the institutionalization of and culture for the use of scientific evidence [28, 30]; (2) encouraging funders to invest in effective evidence communication; and (3) providing alignment between researchers, implementers and policy-makers.

## Discussion

In this rapid scoping review, evidence communication for policy-makers was seen as an innovative approach that leverages specific frameworks to support the communication of evidence to policy makers. Frameworks and their strategies, including tools and techniques for evidence communication were reported, including how to package and synthesize information, and the means to deliver these results to policy-makers. These strategies cater to the needs and preferences of policy-makers, which were analyzed in this scoping review as barriers and facilitators in evidence communication for policy.

This rapid scoping review addressed two important and comprehensive questions to support the discussion on ways to improve the evidence communication to policy-makers, and to support advances in communication processes related to knowledge translation for EIPM. To our knowledge, this is the first study that focuses on a comprehensive view of evidence communication frameworks, guidance, and tools for health policy, while there are knowledge translation frameworks that refer to communication as an integral part, it does not present a comprehensive framework to improve this specific process.

### Evidence communication frameworks

Although communication frameworks to foster the use of evidence are extremely important, this scoping review showed that evidence on this topic is scarce. Only four studies were identified in this review which presented complete communication frameworks [18–21], one of which addresses individual-level decision-makers with a clinical focus [21], which we decided to include in this review as we considered it relevant.

The results of this scoping review reinforce and are reinforced by the results of other previous synthesis studies, in particular those dedicated to systematizing a wide variety of approaches and communication strategies, including KT and communication frameworks, such as the study by Chapman et al. [12].

We also found various strategies (also called “tools” and “techniques” in the included studies) to support evidence communication. While important, these were in the majority of cases not part of structured, integrated frameworks focusing on improving evidence communication for policy.

This scoping review confirmed that effective communication is a complex process going beyond sending messages or making information available. Communication can be understood as the establishment of links between participants in the information exchange process, where communication barriers need to actively addressed and minimized, and participants are willing to interpolate as senders and receivers of relevant and useful messages, in recurrent communication feedback processes.

The findings of this scoping review showed that at times, it is difficult to separate communication from other processes inherent to decision-making such as deliberative processes that are based on dialogues about evidence, if they are built on communication mechanisms. While communication is embedded in and an inherent part of knowledge translation processes, due attention has not been paid to the strategies to more effectively communicate evidence between researchers, evidence intermediaries and policy-makers.

### Categorization of evidence communication strategies

The three groups of communication strategies can be adapted to different contexts, at distinct levels of jurisdiction in policy organizations and health systems. Regarding evidence packaging, evidence syntheses is an important means that the frameworks [5, 12, 18, 19, 23–25, 28–30]. Chapman and colleagues [12] pointed out that evidence syntheses such as evidence briefs for policy proved to be easier to understand, however, there was no difference in effect when compared to systematic reviews in relation to the use, understanding, belief or perceived usefulness of the evidence. Consequently, there is a need to advance knowledge about the impact of communication formats, and to better define the role that evidence synthesis and systematic reviews can play in a complex communication process.

To effectively communicate, targeting and tailoring messages to the audience seems essential. The highest proportion of studies addressing targeting the message, especially of systematic reviews, [5, 12, 20, 30] showed this to be a valued strategy. However, the content of systematic reviews may not be the type of message or information most appreciated by policy-makers, and the use of alternative ways to present them may provide better results [12].

As Chapman and colleagues have already demonstrated [12], combined strategies with multiple interventions to communicate evidence is the strategy for which we found the least amount of evidence, compared e.g. with tailored and targeted messages. This shows that there is still a knowledge gap to be filled, given that the evidence on multicomponent communication strategies is insufficient to make definitive judgements on their effectiveness [12]. Nevertheless, a structured framework on communicating evidence to policy-makers should include a set of strategies that can be combined according to the needs and opportunity of a specific context.

Importantly, when the communication strategy involves stakeholders, motivation and commitment are strengthened. For example, in the context of the use of communication for support formulation and implementation of programs and projects, in the perspective of economic development, meetings are an opportunity to involve participants in a project, and when they are engaged in supporting it, they will do all they can to take it forward [33].

Focus on specific audiences is another strategy that integrated frameworks to support the communication of evidence to policy-makers. These can be divided into Audience segmentation [20], process of dividing audiences into smaller groups that are homogeneous with respect to critical attributes (e.g., demographics, behavior, ideology), and Improve reach of evidence [5, 8, 12, 18–21, 23, 25, 26, 28–30]. Ashcraft et al. [29] showed that communication efforts are more effective when start early and ongoing engagement with policymakers throughout the research process in order to maximize interest and applicability, researchers seek outside support for their work, consider contextual factors (policy-makers’ own personal beliefs and experiences and the prevailing political ideology of a given context), are timely, relevant and accessible. Other studies showed that the use of multiple strategies increases awareness and encourages the use of evidence for decision-making [18, 23] including for policy [19].

Strategies related to information linkage and exchange [12, 18, 20, 28, 29] and person-to-person communication [5, 12, 18, 19, 26, 28–30] showed the contribution of individual or group interaction with a focus on knowledge intermediaries. The interaction of policy-makers with researchers to discuss research results and their implications for practice, including the opportunity to debate implementation strategies, has a profound influence on the use of research evidence as improving recipients’ understanding and hence ability to use and apply evidence (regardless of delivery mode) [12, 18–20, 29].

The categorization of evidence communication strategies based on Langer et al. [20] and McCormack et al. [21] showed a lack of knowledge about implementation strategies for evidence communication, including interventions that could forecast and test future scenarios, which were not found among the included studies.

### Barriers and facilitators on evidence communication

The evidence communication frameworks and strategies found in this scoping review cater for policy-makers' perspectives, needs and preferences. Lasting and trusted relationships between researchers and policy-makers, and the use of adequate channels, language and materials were identified to be a key facilitator. As an example, Dobbins et al. [25] pointed out that policy-makers want to choose and control the information they receive in terms of detail (i.e., abstract, full document) and how the information should be delivered (i.e., electronic, hard copy, Internet). Another study reported the interest and value that policymakers placed on accessing information and interacting directly with researchers, and how this helped to increase willingness to use research evidence in policy [30]. Chapman et al. [12], however, pointed out that implementation of this interaction with researchers did not show a significant effect.

As for the macro-level barriers and facilitators little to no evidence was found.

The diverse and multifaceted nature of communication can influence the communication strategies, their formats and means of implementation for different sectors of public policy, which poses a challenge for developmental communication specialists due to the need for the clarity of information that is shared. The broader function of communication is to create confidence between stakeholders, evaluate the situation, explore options, and seek ample consensus that leads to sustainable change [33].

Global and national initiatives can make strong use of evidence communication to promote evidence-informed policy-making, such as the Evidence-Informed Policy Network (EVIPNet), launched in 2005 by the World Health Organization, whose members produce evidence syntheses and support decision-making processes to help interaction between researchers and policy-makers, and to promote the incorporation of evidence into health policies and programs. In the field of health technology assessment, there are also global collaborative networks such as The International Network of Agencies for Health Technology Assessment (INAHTA), as well as regional and national networks. The Cochrane Collaboration is also recognized as a network that supplies high-quality information through systematic reviews to guide healthcare decision-making. However, this form of access to information was rarely mentioned in the included studies. In addition, guides and checklists, such as Checklist and Guidance for Disseminating Findings from Cochrane Intervention Reviews [34] and Guidelines Policy Influence Plan [35], may be strategies adopted by researchers to communicate and disseminate evidence to improve the understanding and use of evidence in policy. Given this scenario, it is essential that initiatives such as those described above are ready to facilitate, support and encourage the best communication of evidence.

### Limitations

This rapid scoping review has limitations. First, the fact of using studies involving the health sector as an inclusion criterion will have limited relevant information on communication. Second, the searches were undertaken to find only evidence communication frameworks, guidance and tools. A search for studies on knowledge translation, where evidence communication elements can be embedded, could have retrieved more results of interest. Third, although there was no language limitation in the searches, only studies in English were selected, and selection bias could be considered. As this was a rapid review, we must recognize that methodological shortcuts can lead to the loss of some relevant information, but we argue that the risk of other biases has been minimized by our systematic and transparent methods. Fourth, as this is a rapid scoping review, only the title/abstract screening was conducted in duplicate and independently. Fifth, some included studies did not report their methodological design, and we attributed a design classification, based on the described methods, and this may have affected the assessment of the methodological quality of the included studies.

## Conclusions

Communication is essential for knowledge translation and evidence-informed policy-making. More evidence on effectiveness of communication strategies remains needed to advance research evidence communication to policy-makers and stablish comprehensive frameworks that can be more useful than single strategies.

Thus, there is a clear need to increase efforts and investments in identifying and applying suitable strategies for establishing effective evidence communication to policy-makers, in particular on comprehensive communication frameworks as part of knowledge translation processes. Research is also needed on communication for policy that should be piloted to have a more rigorous tool and, at the same time, contribute to knowledge generation on what works. It should be taken into consideration that the group of policy-makers is heterogenous, hence the importance of building long-lasting relationships with researchers and other stakeholders to increase mutual understanding and so that relevant information is available at the time of decision-making.

### Supplementary Information

Additional file 1.

## Data Availability

Available in the supplementary material.
