# Supplementary material for: Research evidence communication for policy-makers: a rapid scoping review on frameworks, guidance and tools, and barriers and facilitators
Source: Health Res Policy Syst. 2024 Aug 8;22:99. doi: 10.1186/s12961-024-01169-9 (PMC11312384; doi:10.1186/s12961-024-01169-9)
Supplement: Supplementary file 1 — Additional file 1. [file 12961_2024_1169_MOESM1_ESM.docx]

Appendix 1. Search strategies

| **Source** | **Date** | **Query** | **Results** |
| --- | --- | --- | --- |
| PubMed | 23/10/2021 | ((("Evidence-Based Medicine"[Mesh] OR "Translational Medical Research"[Mesh]) OR ("Knowledge Translation"[Title/Abstract] OR "Knowledge Translations"[Title/Abstract] OR "Research evidence"[Title/Abstract] OR "Research knowledge"[Title/Abstract] OR "Use of research"[Title/Abstract] OR "Research use"[Title/Abstract] OR "Use of evidence"[Title/Abstract] OR "Knowledge transfer"[Title/Abstract] OR "Knowledge management"[Title/Abstract] OR "Evidence-based"[Title/Abstract] OR "Scientific knowledge"[Title/Abstract])) AND (("Decision Making"[Mesh] OR "Policy Making"[Mesh] OR "Health Policy"[Mesh]) OR ("Decision making"[Title/Abstract] OR "Decision-makers"[Title/Abstract] OR Policymaker*[Title/Abstract] OR "Policy Making" [Title/Abstract] OR Policymaking[Title/Abstract] OR "Health policy"[Title/Abstract] OR "Policy decisions"[Title/Abstract]))) AND (("Communication"[Mesh] OR "Information Dissemination"[Mesh]) OR ("Health communication"[Title/Abstract] OR Communication[Title/Abstract] OR "Information Dissemination"[Title/Abstract] OR "Scientific communication"[Title/Abstract] OR Dissemination[Title/Abstract])) Sort by: Most Recent | 3.855 |
| Embase | 23/10/2021 | #1 'decision making'/exp AND [embase]/lim 316.664  #2 ('policy maker'/exp OR 'policy maker') AND [embase]/lim 371  #3 'health care policy'/exp AND [embase]/lim 139.023  #4 ('decision making':ab,ti OR 'decision-makers':ab,ti OR policymaker*:ab,ti OR 'policy making':ab,ti OR policymaking:ab,ti OR 'health policy':ab,ti OR 'policy decisions':ab,ti) AND [embase]/lim 207.234  #5 'translational research'/exp AND [embase]/lim 16.279  #6 'evidence based medicine'/mj AND [embase]/lim 11.110  #7 ('knowledge translation':ab,ti OR 'knowledge translations':ab,ti OR 'research evidence':ab,ti OR 'research knowledge':ab,ti OR 'use of research':ab,ti OR 'research use':ab,ti OR 'use of evidence':ab,ti OR 'knowledge transfer':ab,ti OR 'knowledge management':ab,ti OR 'evidence-based':ab,ti OR 'scientific knowledge':ab,ti) AND [embase]/lim 139.617  #8 'information dissemination'/exp AND [embase]/lim 13.876  #9 'interpersonal communication'/mj AND [embase]/lim 16.921  #10 ('health communication':ab,ti OR communication:ab,ti OR 'information dissemination':ab,ti OR 'scientific communication':ab,ti OR dissemination:ab,ti) AND [embase]/lim 303.390  #11 #1 OR #2 OR #3 OR #4 509.427  #12 #5 OR #6 OR #7 159.038  #13 #8 OR #9 OR #10 319.377  #14 #11 AND #12 AND #13 | 2.080 |
| SCOPUS | 23/10/2021 | ( KEY ( "Evidence-Based Medicine"  OR  "Knowledge Translation"  OR  "Knowledge Translations"  OR  "Research evidence"  OR  "Research knowledge"  OR  "Use of research"  OR  "Research use"  OR  "Use of evidence"  OR  "Knowledge transfer"  OR  "Knowledge management"  OR  "Evidence-based"  OR  "Scientific knowledge" ) )  AND  ( KEY ("Decision making"  OR  "Decision-makers"  OR  policymaker*  OR  " Policy Making"  OR  policymaking  OR  "Health policy"  OR  "Policy decisions" ) )  AND  ( ( KEY ( "Health communication"  OR  "Information Dissemination"  OR  "Scientific communication"  OR  "Communication research" ) )  OR  ( TITLE (Communication ) ) ) | 1.056 |
| Cochrane Library | 23/10/2021 | #1 MeSH descriptor: [Decision Making] explode all trees 4124  #2 MeSH descriptor: [Policy Making] explode all trees 51  #3 MeSH descriptor: [Health Policy] explode all trees 680  #4 ("Decision making" OR "Decision-makers" OR Policymaker* OR " Policy Making" OR Policymaking OR "Health policy" OR "Policy decisions"):ti,ab,kw 17065  #5 MeSH descriptor: [Evidence-Based Medicine] explode all trees 915  #6 ("Evidence-Based Medicine" OR "Knowledge Translation" OR "Knowledge Translations" OR "Research evidence" OR "Research knowledge" OR "Use of research" OR "Research use" OR "Use of evidence" OR "Knowledge transfer" OR "Knowledge management" OR "Evidence-based" OR "Scientific knowledge"):ti,ab,kw 20218  #7 MeSH descriptor: [Translational Medical Research] explode all trees 124  #8 MeSH descriptor: [Communication] explode all trees 8956  #9 MeSH descriptor: [Information Dissemination] explode all trees 239  #10 ("Health communication" OR Communication OR "Information Dissemination" OR "Scientific communication" OR Dissemination):ti,ab,kw 25958  #11 #1 or #2 or #3 or #4 19247  #12 #5 or #6 or #7 20298  #13 #8 or #9 or #10 30931  #14 #11 and #12 and #13 | 264 |
| VHL Regional Portal | 23/10/2021 | mh:("Evidence-Based Medicine" OR "Translational Medical Research") OR ti:( "Evidence-Based Medicine" OR "Knowledge Translation" OR "Knowledge Translations" OR "Research evidence" OR "Research knowledge" OR "Use of research" OR "Research use" OR "Use of evidence" OR "Knowledge transfer" OR "Knowledge management" OR "Evidence-based" OR "Scientific knowledge") OR ab:("Evidence-Based Medicine" OR "Knowledge Translation" OR "Knowledge Translations" OR "Research evidence" OR "Research knowledge" OR "Use of research" OR "Research use" OR "Use of evidence" OR "Knowledge transfer" OR "Knowledge management" OR "Evidence-based" OR "Scientific knowledge") AND mh:("Decision making" OR "Policy Making" OR "Health Policy") OR ti:( "Decision making" OR "Decision-makers" OR policymaker* OR " Policy Making" OR policymaking OR "Health policy" OR "Policy decisions") OR ab:("Decision making" OR "Decision-makers" OR policymaker* OR " Policy Making" OR policymaking OR "Health policy" OR "Policy decisions") AND mh:("Communication" OR "Information Dissemination") OR ti:("Health communication" OR communication OR "Information Dissemination" OR "Scientific communication" OR dissemination) OR ab:("Health communication" OR communication OR "Information Dissemination" OR "Scientific communication" OR dissemination) AND ( db:("LILACS" OR "BDENF" OR "IBECS" OR "CUMED" OR "PAHOIRIS" OR "PIE" OR "LIS" OR "BIGG" OR "PREPRINT-MEDRXIV") AND mj:("Tomada de Decisões" OR "Medicina Baseada em Evidências" OR "Gestão do Conhecimento" OR "Literatura de Revisão como Assunto" OR "Pesquisa Médica Translacional" OR "Política Informada por Evidências" OR "Planejamento em Saúde" OR "Política de Saúde" OR "Percepção" OR "Formulação de Políticas" OR "Pesquisa")) | 136 |
| Epistemonikos | 23/10/2021 | (Communication OR Dissemination) AND ("Policy Making" OR "Decision making") AND ("Evidence-based" OR "Knowledge Translation" OR "use of evidence") | 64 |
| Health Systems Evidence | 23/10/2021 | (Communication OR Dissemination) AND ("Policy Making" OR "Decision making") AND ("Evidence-based" OR "Knowledge Translation" OR "use of evidence") | 229 |
| Social Systems Evidence | 23/10/2021 | (Communication) AND ("Policy Making" OR "Decision making") AND ("Knowledge Translation" OR "use of evidence") 118 | 118 |
| OpenGrey | 23/10/2021 | (Communication) AND ("Policy Making" OR "Decision making") AND (Evidence) | 35 |
| Google Scholar | 23/10/2021 | (Communication OR Dissemination) AND ("Policy Making" OR "Decision making") AND ("Evidence-based" OR "Knowledge Translation" OR "Use of evidence") - First 10 pages (20 articles each page) | 200 |
| Total | | | 8.037 |

Appendix 2. Full-text articles excluded, with reasons

| **References** | |
| --- | --- |
| **Not about communication to policymakers** | |
| 1 | Ellen ME, Léon G, Bouchard G, Ouimet M, Grimshaw JM, Lavis JN. Barriers, facilitators and views about next steps to implementing supports for evidence-informed decision-making in health systems: a qualitative study. Implement Sci. 2014;9:179. <https://doi.org/10.1186/s13012-014-0179-8> |
| 2 | Erismann S, Pesantes MA, Beran D, Leuenberger A, Farnham A, Berger Gonzalez de White M, et al. How to bring research evidence into policy? Synthesizing strategies of five research projects in low-and middle-income countries. Health Research Policy and Systems. 2021;19(1). <https://doi.org/10.1186/s12961-020-00646-1> |
| 3 | Guetat S, Dakhli SBD. The complementary roles of information systems and knowledge management systems: A framework based on Popper’s three worlds theory. Le Mans University, Le Mans, France. Communications in Computer and Information Science. 2010;109:374–384. <https://doi.org/10.1007/978-3-642-16402-6_39> |
| 4 | Guldbrandsson K, Stenström N, Winzer R. The DECIDE evidence to recommendation framework adapted to the public health field in Sweden. Health Promot Int. 2016;31(4):749-754. <https://doi.org/10.1093/heapro/dav060> |
| 5 | Hanney SR, Kanya L, Pokhrel S, Jones TH, Boaz A. How to strengthen a health research system: WHO's review, whose literature and who is providing leadership? Health Res Policy Syst. 2020;18(1):72. <https://doi.org/10.1186/s12961-020-00581-1> |
| 6 | Harris JK. Communication ties across the national network of local health departments. Am J Prev Med. 2013;44(3):247-53. <https://doi.org/10.1016/j.amepre.2012.10.028> |
| 7 | Karamitri I, Talias MA, Bellali T. Knowledge management practices in healthcare settings: a systematic review. Int J Health Plann Manage. 2017;32(1):4-18. <https://doi.org/10.1002/hpm.2303> |
| 8 | Lillefjell M, Knudtsen MS, Wist G, Ihlebæk C. From knowledge to action in public health management: experiences from a Norwegian context. Scand J Public Health. 2013;41(8):771-7. <https://doi.org/10.1177/1403494813496600> |
| 9 | Lin C, Li L, Lee SJ, Chen L, Pan Y, Guan J. Using Conjoint Analysis to Investigate Hospital Directors' Preference in Adoption of an Evidence-based Intervention. Int J Healthc Manag. 2021;14(2):328-334. <https://doi.org/10.1080/20479700.2019.1645925> |
| 10 | Oxman AD, Vandvik PO, Lavis JN, Fretheim A, Lewin S. SUPPORT Tools for evidence-informed health Policymaking (STP) 2: Improving how your organisation supports the use of research evidence to inform policymaking. Health research policy and systems. 2009;7:S2–S2. <https://doi.org/10.1186/1478-4505-7-S1-S2> |
| 11 | Redman S, Turner T, Davies H, Williamson A, Haynes A, Brennan S, et al. The SPIRIT Action Framework: A structured approach to selecting and testing strategies to increase the use of research in policy. Social science & medicine (1982). 2015;136:147–55. <https://doi.org/10.1016/j.socscimed.2015.05.009> |
| 12 | Reitmanova S. Knowledge translation in health research: a novel approach to health sciences education. Medical education online. 2009;14:10–10. <https://doi.org/10.3885/meo.2009.T0000142> |
| 13 | Yearwood A, Bachan N. Evidence use, public participation, and multi-sectoral collaboration, during the COVID-19 pandemic: A Case study of Trinidad and Tobago. SSRN. 2021. <https://doi.org/10.13140/RG.2.2.35809.22881> |
| 14 | Yost J, Dobbins M, Traynor R, Decorby K, Workentine S, Greco L. Tools to support evidence-informed public health decision making. BMC Public Health. 2014;14(1). <https://doi.org/10.1186/1471-2458-14-728> |
| 15 | Wei Y, Pong RW, Shi L, Ming J, Tang M, Mao Y, et al. Perceptions of health technology assessment knowledge translation in China: A qualitative study on HTA researchers and policy-makers. International Journal of Healthcare Technology and Management. 2017;16(1):44–58. <https://doi.org/10.1504/IJHTM.2017.10008509> |
| 16 | Parmelli E, Amato L, Saitto C, Davoli M; Gruppo di Lavoro "DECIDE Italia. DECIDE: uno strumento per rendere trasparenti i criteri utilizzati per le decisioni in sanità [DECIDE: developing and evaluating communication strategies to support informed decisions and practice based on evidence]. Recenti Prog Med. 2013;104(10):522-31. Italian. <https://doi.org/10.1701/1349.14997> |
| **Focus on Knowledge Translation** | |
| 17 | Dobbins M, Ciliska D, Cockerill R, Barnsley J, DiCenso A. A framework for the dissemination and utilization of research for health-care policy and practice. Online J Knowl Synth Nurs. 2002;9:7. <https://doi.org/10.1111/j.1524-475X.2002.00149.x> |
| 18 | Deutsche Gesellschaft für Technische Zusammenarbeit. Strategic Communication for Sustainable development: A Conceptual Overview. 2006. <https://www.cbd.int/cepa/toolkit/2008/doc/strategic%20communication%20for%20sustainable%20development.pdf> |
| 19 | Hanney, S., Kanya, L., Pokhrel, S., Jones, T., & Boaz, A. What is the evidence on policies, interventions and tools for establishing and/or strengthening national health research systems and their effectiveness? Copenhagen: WHO Regional Office for Europe; 2020b (Health Evidence Network (HEN) synthesis report 69). <https://apps.who.int/iris/handle/10665/331703> |
| 20 | Househ M, Kushniruk A, Cloutier-Fisher D, Carleton B. Technology enabled knowledge exchange: development of a conceptual framework. J Med Syst. 2011 Aug;35(4):713-21. <https://doi.org/10.1007/s10916-009-9408-0> |
| 21 | Hyder AA, Corluka A, Winch PJ, El-Shinnawy A, Ghassany H, Malekafzali H, Lim MK, Mfutso-Bengo J, Segura E, Ghaffar A. National policy-makers speak out: are researchers giving them what they need? Health Policy Plan. 2011;26(1):73-82. <https://doi.org/10.1093/heapol/czq020> |
| 22 | Mefalopulos, P. Development communication sourcebook: Broadening the boundaries of communication. World Bank Publications, 2008. <https://documents1.worldbank.org/curated/en/752011468314090450/pdf/446360Dev0Comm1ns0handbook01PUBLIC1.pdf> |
| 23 | Orem JN, Mafigiri DK, Marchal B, Ssengooba F, Macq J, Criel B. Research, evidence and policymaking: the perspectives of policy actors on improving uptake of evidence in health policy development and implementation in Uganda. BMC Public Health. 2012 Feb 9;12:109. <https://doi.org/10.1186/1471-2458-12-109> |
| 24 | Tiwari BB, Ban A, Gurung S, Karki KB. Translating evidence into policy: opinions and insights of Health Researchers and Policymakers in Nepal. BMC Health Serv Res. 2021;21(1):1066. <https://doi.org/10.1186/s12913-021-07102-y> |
| 25 | Yost J, Mackintosh J, Read K, Dobbins M. Promoting Awareness of Key Resources for Evidence-Informed Decision-making in Public Health: An Evaluation of a Webinar Series about Knowledge Translation Methods and Tools. Front Public Health. 2016 Apr 22;4:72. <https://doi.org/10.3389/fpubh.2016.00072> |
| 26 | Zhelev Z, Garside R, Hyde C. A qualitative study into the difficulties experienced by healthcare decision makers when reading a Cochrane diagnostic test accuracy review. Syst Rev. 2013 May 16;2:32. <https://doi.org/10.1186/2046-4053-2-32> |
| **Does not refer to the health sector** | |
| 27 | Campbell, S., Benita, S., Coates, E., Davies, P. & Penn, G. Analysis for Policy: Evidence based Policy and Practice. London: HM Treasury, Government Social Research Unit. 2007. <https://issuu.com/cecicastillod/docs/pu256_160407> |
| 28 | Cherney A, Head B, Povey J, Ferguson M, Boreham P. Use of Academic Social Research by Public Officials: Exploring Preferences and Constraints That Impact on Research Use. Evid Policy. 2015;11(2):169–88. <http://dx.doi.org/10.1332/174426514X14138926450067> |
| 29 | Cochrane. Checklist and Guidance For disseminating findings from Cochrane intervention reviews. 2020. <https://training.cochrane.org/sites/training.cochrane.org/files/public/uploads/Checklist%20FINAL%20version%201.1%20April%202020pdf.pdf> |
| 30 | International Initiative for Impact Evaluation. Guidelines POLICY INFLUENCE PLAN. <https://www.conafe.gob.mx/transparencia1/libro-blanco/2-anexos-edu-inicial/anexo99/MX%20-%20OW3-1079%20-Policy%20Influence%20Plan%20-%20Revised%202011-06-13%20(2).pdf> |
| 31 | Paine Cronin G, Sadan M. Use of evidence in policy making in South Africa: An exploratory study of attitudes of senior government officials. African Evaluation Journal. 2015;3(1):1-10. <https://doi.org/10.4102/aej.v3i1.145> |
| 32 | Pulido-Salgado M, Castaneda Mena FA. Bringing Policymakers to Science Through Communication: A Perspective From Latin America. Front Res Metr Anal. 2021;6:654191. <https://doi.org/10.3389/frma.2021.654191> |
| 33 | Pullin AS, Knight TM. Support for decision making in conservation practice: an evidence-based approach. Journal for Nature Conservation. 2003;11(2):83–90. <https://doi.org/10.1078/1617-1381-00040> |
| **Focus on evidence use/access** | |
| 34 | Pettman TL, Hall BJ, Waters E, de Silva-Sanigorski A, Armstrong R, Doyle J. Communicating with decision-makers through evidence reviews. J Public Health (Oxf). 2011;33(4):630-3. <https://doi.org/10.1093/pubmed/fdr092> |
| 35 | Moore G, Redman S, Rudge S, Haynes A. Do policy-makers find commissioned rapid reviews useful? Health Res Policy Syst. 2018;16(1):17. <https://doi.org/10.1186/s12961-018-0293-1> |
| 36 | Uneke CJ, Ezeoha AE, Uro-Chukwu H, Ezeonu CT, Ogbu O, Onwe F, Edoga C. Improving Nigerian health policymakers' capacity to access and utilize policy relevant evidence: outcome of information and communication technology training workshop. Pan Afr Med J. 2015;21:212. <https://doi.org/10.11604/pamj.2015.21.212.6375> |
| 37 | Williamson A, Makkar SR, McGrath C, Redman S. How Can the Use of Evidence in Mental Health Policy Be Increased? A Systematic Review. Psychiatr Serv. 2015;66(8):783-97. <https://doi.org/10.1176/appi.ps.201400329> |
| **Focus on clinical practices** | |
| 38 | Kreps GL. Applying Weick's model of organizing to health care and health promotion: highlighting the central role of health communication. Patient Educ Couns. 2009;74(3):347-55. <https://doi.org/10.1016/j.pec.2008.12.002> |
| 39 | Shahmoradi L, Safadari R, Jimma W. Knowledge Management Implementation and the Tools Utilized in Healthcare for Evidence-Based Decision Making: A Systematic Review. Ethiop J Health Sci. 2017;27(5):541-558. <https://doi.org/10.4314/ejhs.v27i5.13> |
| **Not about policymakers** | |
| 40 | Winterbottom A, Bekker HL, Conner M, Mooney A. Does narrative information bias individual's decision making? A systematic review. Soc Sci Med. 2008;67(12):2079-88. <https://doi.org/10.1016/j.socscimed.2008.09.037> |
| **Focus on implementation** | |
| 41 | Sarkies MN, Bowles KA, Skinner EH, Haas R, Lane H, Haines TP. The effectiveness of research implementation strategies for promoting evidence-informed policy and management decisions in healthcare: a systematic review. Implement Sci. 2017;12(1):132. <https://doi.org/10.1186/s13012-017-0662-0> |

Appendix 3. Detailed characteristics of the included studies.

| Study (in alphabetical order) | Objective | Results | | | Conclusion | Limitations | Gaps/Lacks |
| --- | --- | --- | --- | --- | --- | --- | --- |
|  |  | Packaging tools | Dissemination and Communication tools | Perception of policymakers |  |  |  |
| Ashcraft et al. 2020 [29] | To synthesize existing evidence about how research has been used to influence social policy. | **Information packaging and synthesis tools:** Summary of research; policy briefs, original legal research article; literature reviews and summary reports; County Health Rankings Report  **Visualization and modelling tools:** Brochures, fact sheets and press releases | **Electronic tools for dissemination and communication:** Electronic and hard copy print materials  **Tools for automated electronic dissemination of information:** Media outreach campaign, via websites, email listserv and social media  **Tools for person-to-person communication of health information:** Person-to-person communication; conferences and webinars; Public education campaign; small-group meetings; Congressional committee hearings and legislative development  **Information linkage and exchange tools:** evidence champions or brokers | **Facilitators:**  - While all styles of brief were considered understandable and credible, opinions on the usefulness of the brief varied by the style of the brief and by the level of policymaker. These findings suggest that targeted, audience specific research evidence materials may be more likely to be used by policymakers than generic research evidence.  - One study explored the usefulness of electronic vs. printed research material and again found differences by type of policymaker—legislators were more likely to read hard copy printed material, while staffers gave higher ratings to online content. Not surprisingly, the age of the policymaker also played a role in the choice to access electronic or printed material, with younger policymakers much more likely to read electronic copy than were their older peers.  - The most useful research is that which is consistent and specific to the needs of the policymaker  **Barriers:**  - Often, policymakers appear unwilling or uninterested in considering the application of evidence to their work  - A lack of relevant high quality or conclusive research  **Perspectives:**  - Early and ongoing engagement with policymakers throughout the research process  - The need for researchers to garner outside support for their work | The most frequently referenced channel for dissemination to policymakers was print materials, with personal communication (including both in-person and electronic meetings and individual communications) a close second. Corresponding strategies for effective dissemination to policymakers included starting early, drumming-up support, using champions and brokers, understanding the context, ensuring timeliness, relevance, and accessibility of research products, and knowing the players and the process. A shared feature of these strategies is the distillation of complex research findings into accessible pieces of relevant information that can then be delivered via multiple avenues. Engaging policymakers early as contributing members of the research team, maintaining communication during the research process, and presenting relevant findings in a clear, concise manner may empower both researchers and policymakers to further apply scientific evidence to improve social policy in the United States. | First, some studies might have been missed. Second, the article provides a rationale for focusing on US studies and that its findings may not be generalizable to other countries. Third, the authors were unable to assess the risk of bias for individual studies as current standards note difficulties in assessing quality and bias in qualitative research. Fourth, many studies examined multiple channels or strategies for how research influences policy, so the parsing of singular strategies (e.g., policy brief, in-person meeting) as an effective approach should be interpreted with caution. One significant challenge to increasing the rigor in dissemination research studies is the difficulty in choosing and then measuring an outcome. Many of the studies included in this review are either case studies or descriptive, making it difficult to determine what, if any, impact the given research had on policy. | Not reported |
| Blessing et al. 2017 [18] | This review examined methods to support the use of health information by public health and health system policy-makers, asking the question: “What is the evidence on mechanisms and tools for use of health information for decision-making?” | **Information packaging and synthesis tools:** Policy briefs; public health reports, Local health memoranda (or messages)  **Visualization and modelling tools:** Graphs and charts (line graphs, bar charts or pie charts), infographics, data dashboards, dynamic graphs; maps and e-atlases; local health memoranda (or messages) | **Communication and dissemination tools:** Modelling and simulation tools;  Public health surveillance programmes  **Electronic tools for dissemination and communication:** Public health surveillance programmes; National clinical databases  **Tools for automated electronic dissemination of information:** Newsletters, email messages, tweets or phone messages  **Tools for person-to-person communication of health information:** personal contacts; oral presentations, discussions and deliberative dialogues; Health forum; Conferences; workshops  **Information linkage and exchange tools:** evidence champions or brokers; individual knowledge brokers; Stakeholder networks | Not reported | Knowledge translation mechanisms and tools that support the use of health information for policy decision-making have been presented, categorized and linked to intended outcomes for users. A taxonomy of four broad categories indicates the ways in which health information can be taken up by policy-makers: packaging tools, application tools, dissemination and communication tools, and linkage and exchange tools. Health information is a key component of any health information system, together with active stakeholders, the end-users of health information (policy/decision-makers), knowledge brokers and networks, and health information producers. Mechanisms and tools can be used either through push efforts, where health information producers collect, analyze and make available data on their own initiative, or pull efforts, where policy-makers request specific health information to meet certain policy needs. The most productive scenario, however, is characterized by exchange efforts for information, where stakeholder groups for both the generation and the usage areas are engaged in a real partnership, often facilitated by knowledge brokers. This approach means that the prospects are higher for producing and using relevant and timely health information in policy formulation. Tools and mechanisms that work best in a given context can become integrated in organizational processes and the broader health system. The use of health information may be enhanced by applying packaging tools, including policy briefs and visualization tools. Application tools provide an additional layer of knowledge to existing health information by forecasting and testing future scenarios. Finally, active dissemination and communication of health information is as important as producing and packaging the actual data. | This review has several limitations. First, systematic literature review methodology was unable to identify all the latest innovations and practices from peer-reviewed literature because not all available tools and cutting-edge approaches are formally published. Second, there was a bias towards English language literature because of the greater representation of such publications in PubMed and Scopus. Third, it was not possible to explicitly appraise methodologies or qualities across the studies included in this review as studies were primarily of a descriptive nature. Finally, there was a gap in evidence regarding tools specifically used for incorporating health information into policy-making as well as for achieving behavior change among policy-makers and health information producers. This may be because most authors do not make a distinction between tools or mechanisms for evidence in general and those for health information. In addition, information on how policy-makers actually employ knowledge translation tools and mechanisms to generate the intended outcomes was also limited. Hence, it was difficult to comprehensively list the support (e.g. technical, organizational, administrative, legislative, infra structural) required to implement the mechanisms and tools. | Not reported |
| Campbell et al. 2009 [30] | To explore the views and current practice of both policy makers and researchers about the use of evidence in policy. Specifically, the study aimed to: i. Describe the extent to which policy makers and researchers believed that research is currently used to inform policy; ii. Investigate current practice in relation to the dissemination of and access to research findings for policy; iii. Explore the extent of communication and exchange between researchers and policy makers; iv. Examine incentives for increasing the use of research in policy. | **Information packaging and synthesis tools:** Summaries of local data, brief research summaries, systematic reviews | **Tools for person-to-person communication of health information:** Forums, Conference, informal approach (from researchers) to provide a research perspective on a policy issue  **Information linkage and exchange tools: A**ctive participation in a research team, advisory processes (e.g.: policy development committee); collaborative approaches; one-to-one consultations; Informal approaches from researchers | **Facilitators:**  - Summaries of local data were rated as highly useful by 78% of these respondents, with 72% rating brief research summaries and 64% rating systematic reviews as highly useful respectively.  - Of those (policymakers) who had wanted to discuss ideas with a researcher in the previous year, 57% (42% of the total sample) were easily able to contact a relevant researcher when needed.  - Eighteen percent of the sample had collaborated on a successful competitive research grant.  **Barriers:**  - Most of policymakers believed that the research was not presented in a useful way.  - It was evident that policy makers needed all three types of synthesis (synthesis of local data, brief research summary, systematic reviews) but had difficulty finding brief research summaries and systematic reviews when they were needed.  - Half of the interviewees reported active participation in a research team. However, fewer had been involved in the sorts of activities that are likely to facilitate communication and application of research, such as participating in the analysis, writing up and publication of the research results.  - Most common reasons for not using research in policy were: the absence of appropriate and/or relevant research (29%); a lack of skills or capacity to access or acquire relevant research (24%); the need to consider local agendas and other policy drivers (24%); and time pressures (21%).  - Fifty five percent of the respondents were not aware of a NSW Health guideline that required evidence to be checked during policy development.  **Perspectives:**  - The most frequently nominated strategy for improving the use of research in respondents' organizations was improved access to research and researchers (32%). Participants' suggestions included: "building bridging systems between researchers and policy makers" and "standing arrangement with key research groups and key research people who can readily assist in policy making".  - Policy makers felt that organizational reinforcement for evidence-informed policy could be improved. | These findings suggest four strategies to assist in increasing the use of research in policy: making research findings more accessible to policy makers; increasing opportunities for interaction between policy makers and researchers; addressing structural barriers such as research receptivity in policy agencies and a lack of incentives for academics to link with policy; and increasing the relevance of research to policy. | Not reported | Not reported |
| Chapman et al., 2021 [12] | To evaluate the effectiveness of knowledge communication and dissemination interventions, strategies or approaches targeting policy-makers and health managers. | **Information packaging and synthesis tools:**  Evidence summaries; systematic review. There was little to no difference in the effect of evidence summaries compared with complete systematic review on the use, understanding, belief or perceived usefulness of evidence. However, summary of findings tables and graded entry summaries were perceived as slightly easier to understand than complete systematic review  **Visualization and modelling tools:**  -The use of tailored and targeted messages (both strategies are usually implemented together) based on reliable evidence seems to lead to changes not only in knowledge but also in decision-making practices of the target audience  -Eight studies used “physical or printed tools”, for example, drawing, painting, research summaries, journal prints and bulletins;  - The evidence regarding arts-based interventions and multicomponent communication strategies was insufficient to make definitive judgements about their effectiveness  - Tree studies reported the use of “media or social media”: magazines and journal prints, booklets with testimonies, advocacy summaries of personal experiences | **Electronic tools for dissemination and communication:** Platforms for sharing health information; Websites; web-based information and communication, dissemination of systematic reviews through the website; Web-based training programs, structured seminar series; Repository of systematic reviews; database access; Media or social media (media and public opinion, television appearances, entertainment education - prime-time network TV storyline, short films, wikis, blogs and online forums)  **Tools for automated electronic dissemination of information:** Targeted messaging; Series of emails with links to full references, abstracts and summaries.  **Tools for person-to-person communication of health information:** conference technology; conference technology to support knowledge sharing; structured  seminar series; Interventions to enhance recipients’ ability to use and apply evidence (e.g. training workshops with an interactive component); narrative action reflection workshops; face to face interactions  **Information linkage and exchange tools:**  Interventions designed to motivate target audiences to use and apply evidence (e.g. knowledge brokering); individual knowledge brokers; knowledge translation platforms; organization-wide capacity-development initiatives; research engagement actions and multifaceted dissemination strategies; Communities of practice; networks; Interactions and interpersonal connections with staff, tailored exchanges within and across departments and disciplines; Grant-funded collaboration involving policy-makers | **Facilitators:**  Facilitators related to the format and content of synthesis documents, for instance the inclusion of a format with graded access and executive summary.  **Barriers:**  Nine studies reported barriers to the communication and dissemination processes.  -At the individual level, the lack of time to be trained or to critically read articles, and the lack of awareness, familiarity and perceived usefulness of research and motivation were mentioned.  - At the organizational level, the lack of financial or adequate infrastructure, frequent staff turnover, the health system in general, the lack of access to evidence, the lack of leadership, an organizational culture not conducive to communication and dissemination processes, and difficulty in establishing alliances or developing a shared vision were identified. Scarce training of human resources and peer support was a specific barrier mentioned with regard to human resources.  - barriers related to research itself included the type of research being considered, the need to refine methodologies used for conducting reviews, the need to consider diverse contexts, the perceived credibility of findings, timeliness of research and the relevance of research in day-to-day decision-making.  **Perspectives:**  This complexity requires that strategies go beyond the simple “push” or “pull” of research evidence (communication and dissemination), combining them with strategies promoting processes of co-production of knowledge, where decision-makers and researchers are interacting from the beginning, increasing the probability of translating research into policy. These multifaceted approaches include capacity-building workshops, policy dialogues or use of knowledge brokers, implemented through different structures such as KTPs. | Knowledge communication strategies based on the use of targeted and tailored messages, and knowledge dissemination strategies that not only enhance recipients’ ability to use and apply evidence but also motivate them to do it, seem to be the more effective in promoting the use of evidence by managers and policy-makers. Passive dissemination strategies aimed only at increasing the access and reach of the evidence does not seem to have a relevant effect. | - Took a “rapid overview” approach, implying that only part of the study selection and data extraction processes were performed in duplicate, which could have introduced bias and potential errors in our overview.  We did not find an established intervention classification in the field, we chose to adapt one previously developed by others. Because of the lack of standards regarding precise definitions of interventions and outcomes, we relied on our individual judgements when classifying the type of interventions and outcomes that each included review was focused on.  Because most of the evidence is presented in a narrative format, we were unable to evaluate the certainty of the evidence with established methodological approaches (such as GRADE), and we used an alternative approach that relied heavily on our judgements about the amount and strength of the evidence available in each review. | The need for more robust studies, especially experimental studies measuring the effectiveness of different communication and dissemination strategies and programs in different settings, including hypothetical scenarios, were featured in many of them. Others reported the need to evaluate specific strategies such as the use of knowledge brokers, KTPs, different forms of narratives or art. One study reported the need for cost-effectiveness studies in KT strategies.  Highlighted the need for qualitative studies to better understand the knowledge, beliefs and attitudes of policy-makers towards narratives or their attitudes towards research in general, user needs, and individual attributes and contextual factors that could influence the effectiveness.  - The description of the intervention. |
| Dobbins et al. 2004 [25] | To Discover public health decision makers’ preferences for content, format, and channels for receiving research knowledge, so as to begin development of a comprehensive national public health knowledge transfer strategy. | **Information packaging and synthesis tools:** Summary statement | **Electronic tools for dissemination and communication:**  Hard copy; electronic copy; Online/electronic service | **Needs:**  - Generally, the summary statement was well received by participants.  - credibility, quality of research, timeliness, reliability, customizability, applicability, accessibility, electronic linkages, and knowledge management all attempt in some way to minimize the time required to actually incorporate research evidence within the decision-making process.  - It was especially important to decision makers that electronic sources run smoothly and had links that always worked as expected.  - participants perceived quality to be high when the information was well written, concise, easily understood, well organized, easy to scan quickly, and linked to other relevant, high-quality documents.  - Quality of information was also linked with credibility and reliability. Several factors determined the quality of research information: had to be current and provide a publication date; was framed within the local, provincial, or national context; was jargon-free and transparent; reported what worked and what didn’t; listed recommendations ranked in order of effectiveness; included cost analyses.  - The timing for receiving research evidence was also identified as an important aspect of information quality. Decision makers were more receptive to receiving and using research evidence when it related directly to issues on which they were currently working.  - Access to additional information, such as the full document, had to occur smoothly and quickly to be of use to decision makers. It was very important to decision makers that they received information only in their area(s) of interest so that most or all of what they received was relevant to their particular role(s).  - Electronic format was the preference of the majority, but there were still instances in which decision makers wanted a hard copy—for a very long document or when they needed to post/share a piece of information.  **Facilitators:**  - Generally, the summary statement was well received by participants.  - credibility, quality of research, timeliness, reliability, customizability, applicability, accessibility, electronic linkages, and knowledge management all attempt in some way to minimize the time required to actually incorporate research evidence within the decision-making process.  - It was especially important to decision makers that electronic sources run smoothly and had links that always worked as expected.  - participants perceived quality to be high when the information was well written, concise, easily understood, well organized, easy to scan quickly, and linked to other relevant, high-quality documents.  - Quality of information was also linked with credibility and reliability. Several factors determined the quality of research information: had to be current and provide a publication date; was framed within the local, provincial, or national context; was jargon-free and transparent; reported what worked and what didn’t; listed recommendations ranked in order of effectiveness; included cost analyses.  - The timing for receiving research evidence was also identified as an important aspect of information quality. Decision makers were more receptive to receiving and using research evidence when it related directly to issues on which they were currently working.  - Access to additional information, such as the full document, had to occur smoothly and quickly to be of use to decision makers. It was very important to decision makers that they received information only in their area(s) of interest so that most or all of what they received was relevant to their particular role(s).  - Electronic format was the preference of the majority, but there were still instances in which decision makers wanted a hard copy—for a very long document or when they needed to post/share a piece of information.  **Barriers:**  - Decision makers indicated that accessibility to information sources continued to be a barrier. For example, a number of decision makers indicated they did not have ready access to the Internet and did not have access to other technology such as high-speed printers. In addition, access to librarians, and others (research assistants/consultants) to assist with searching, retrieval, and interpretation varied widely across the country, and generally there were little to no funds for such roles within most organizations.  - Decision makers indicated that they received information from a variety of sources and that they were overwhelmed with the effort to effectively catalogue and assimilate it.  **Perspectives:**  - Decision makers suggested that researchers aim to stay on top of priority issues across the country and dispatch information according to local priorities.  - It was clear from the data that decision makers wanted choices and control over the information they received in at least two distinct ways: the amount of detail they received (i.e., abstract, summary, full document) and how information was delivered (i.e., electronic/PDF, hard copy, Internet). | The data demonstrated that in addition to providing decision makers with relevant and timely research evidence, a KT strategy must also provide the information in a reliable and consistent way, and must give decision makers options for customizing how the information will be received. | The summary statement created was written for a review in which the topic area was tobacco use reduction. Therefore, it is possible that focus groups attracted individuals whose main interests centered in tobacco use reduction, and that views expressed were not representative of the larger public health and health promotion sector. Though it raises the question of whether there are topic areas within public health to which findings may not be transferable, attempts were made by the moderator to keep discussions on a conceptual level during the focus groups. Finally, focus groups were conducted in English, which may have excluded potential French-speaking participants who may otherwise have been interested in taking part. | Not reported |
| Dobbins et al. 2007 [5] | This article is specifically focused on describing public health decision-makers' definition of evidence-based decision-making (EBDM), as well as their informational needs and preferences for receiving research evidence. | **Information packaging and synthesis tools:**  Summaries, executive summary  **Visualization and modelling tools:** Newsletters containing summaries of current research developed and directly e-mail | **Electronic tools for dissemination and communication:** They also expressed interest in using the Internet to access relevant research evidence and suggested that reports could be either distributed through public health professional organizations or through a clearinghouse.  **Linkage and exchange tools:**  one-to-one interaction with the researcher to discuss research findings | **Perspectives:**  - It is clear from this study that ongoing efforts are required to effectively transmit the key messages from systematic reviews using various communication channels and products.  - In addition to these content issues, additional efforts are needed to address issues such as relevance, implications for practice, and implementation strategies.  - Decision-makers are looking to researchers to not only describe the facts (findings from research studies), but also to provide guidance and suggestions concerning implementation of the findings into program planning and practice. They are specifically interested in reading about what works and what doesn’t work in which instances and for which populations.  - The consensus among respondents of the importance and value of an executive summary indicates the need for researchers to dedicate more time and effort in writing these summaries.  - Careful thought should be given to the content of the summary, the key messages that can and should be highlighted, and the identification of potential practice and policy implications. | Consensus exists among Ontario public health decision-makers about the definition of evidence-based decision-making. This is a promising finding particularly because it shows the extent to which the use of research evidence is being integrated into public health decision-making, at least at the program planning level. | The number of respondents in this study was small, and there is likely more variability across all public health professionals in Ontario than was observed in this study. | Not reported |
| Funk et al. 2021 [19] | The aim of developing this framework was to collate and integrate relevant existing KT and policy frameworks, along with the data from the HI specific literature from the review, to increase the acceptability of HI in policy-making. It can further assist decision-makers to understand where and how HI can fit into the policy process. | **Information packaging and synthesis tools:**  Policy briefs or evidence-briefs for policy  **Visualization and modelling tools:**  Modelling tools; publicly-accessible platform (included geographical maps); memos to/from the government | **Electronic tools for dissemination and communication:** Data platforms included in the literature were big data platforms, platforms where large amounts of data are stored with open-access, interactive data platforms, or surveillance platforms.  Communication and dissemination tools can cover all four knowledge translation mechanisms, depending on what exact tool is chosen.  **Tools for automated electronic dissemination of information:** Letters and memos  **Tools for person-to-person communication of health information:** oral presentations, where one or several persons can present health information to  a specific audience, such as policy-makers | Not reported | We found that different KT tools exist, which can help bridge the HI gap to policy-makers. Our framework provides an overview of how the KT processes of HI are likely to occur and how communication and dissemination tools or information linkage and exchange tools are necessary for the HI to reach the policy-cycle, founded on other type of KT tools. It is important to consider that the mere use of individual KT tools is not enough for successful EIP, and for successful translation of HI. KT tools must be used properly, requiring good knowledge of the audience and context and often through a multi-tool approach. Monitoring and evaluation of such KT processes needs to be in place in order to test the proposed framework in view of improving EIP prospectively and in the long run. It is essential to establish frameworks that integrate HI, research, and experience among those who make decisions in health policies for a more effective political response. | We used the same search strategy as was used in the HEN report, which enabled us the comparability with the work done previously and inclusion of the report's findings into this study. However, this also meant we were limited by that search strategy and did not include more specified terminology or search strings | Not reported |
| Innvaer et al. 2002 [27] | To summarize the evidence from interview studies of facilitators of, and barriers to, the use of research evidence by health policy-makers. | Not reported | Not reported | **Facilitators:**  - Personal contact between researchers and policy-makers.  - Timeliness and relevance of the research.  - Research that included a summary with clear recommendations.  - Good quality research.  - Research that confirmed current policy or endorsed self-interest.  - Community pressure or client demand for research.  - Research that included effectiveness data.  Based on the findings of these studies, personal two-way communication between researchers and decision-makers should be used to facilitate the use of research. This can reduce mutual mistrust and promote a better understanding of policy-making by researchers and research by policy-makers. It can inform researchers about what the decision-makers consider timely, relevant questions and policy-makers about how to obtain valid answers to these questions. However, the frequently identified facilitators, including personal two-way communication, may not be easy to establish – for example, because of political instability or high turnover of policy-making staff.  **Barriers:**  - Absence of personal contact between researchers and policy-makers.  - Lack of timeliness or relevance of research.  - Mutual mistrust, including perceived political naivety of scientists and scientific naivety of policy-makers.  - Power and budget struggles.  - Poor quality of research.  - Political instability or high turnover of policy-making staff. | Interview studies with health policy-makers provide only limited support for commonly held beliefs about facilitators of, and barriers to, their use of evidence, and raise questions about commonsense proposals for improving the use of research for policy decisions. Two-way personal communication, the most common suggestion, may improve the appropriate use of research evidence, but it might also promote selective (inappropriate) use of research evidence.  Researchers who wish to increase the use of the results of their research should: have personal and close two-way communication with decision-makers; provide decision-makers with a brief summary of their research with clear policy recommendations; ensure that their research is perceived as timely, relevant and of high quality; include effectiveness data; argue that the results of their research are relevant to current policy and demands from the community. They should avoid getting involved in power and budget struggles and be aware of the high turnover of policy-making staff. | The limitations of the literature we reviewed:  - indexed in electronic databases  - Quality studies  - Contact with researchers  - Diversity of contexts in which health policy decision-making occurs  - No factor was mentioned in more than 13 of the 24 studies as a facilitator or barrier  - Different definitions for the term "use of evidence" | Future research should combine interviews with document analysis, focus on commissioned research and clearly de ne what is meant by ‘use’ of research. |
| Langer et al. 2016 [20] | The aim of this research project is to review the evidence-base relevant to increasing the use of research evidence by decision-makers; in other words, to review one aspect of the science of using scientific knowledge. | **Information packaging and synthesis tools:**  - Personalized and targeted manner of evidence communication.  - Capability, motivation, and opportunity (CMO): targeted communication, audience segmentation in dissemination, and more accessible and user-friendly packaging of evidence.  - Explaining uncertainty: regularly applying techniques to explain uncertainty to decrease ambivalence in research results.  - Narratives: enhancing existing evidence communication practices to increase the relevance and accessibility of research results.  **Visualization and modelling tools:**  - Audio recordings of research summaries for health practitioners to listen to while driving that did not identify a positive effect of applying communication & access  - Tailoring & targeting: regularly applying tailoring & targeting to align communication of evidence to decision-makers’ professional needs & personal preference.  - Framing (gain/loss): aligning the communication of the research results with the cognitive characteristics of the decision or the desired behavior.  - Framing (norms / identities): aligning the communication of evidence or the concept of EIDM with the decision-makers’ existing norms and identity.  - Audience segmentation: fitting EIDM promotion / research message to decision-maker audience  - Information design: increasing the accessibility as well as visual appeal of evidence | **Automated electronic methods:**  - Reminders, incentives, framing and anchoring  **Information packaging and synthesis tools:**  - Systematic review reports in a more user-friendly manner (see above for related evidence on this) with on-demand service hotlines that decision-makers can consult when in need of evidence.  - Systematic review databases, and training courses on skills related to EIDM (evidence-informed decision-making)  **Information linkage and exchange tools:**  - Motivation to use evidence was created through a more personalized and targeted manner of evidence communication. This entailed approaching decision-makers prior to the conduct of the research to seek their permission for a future dissemination as well as sending weekly, targeted messages that advised decision-makers of articles in a registry relevant to their program area. These motivation building components were then coupled with an opportunity to use evidence. This opportunity included providing decision-makers’ with access to an evidence portal and systematic review summaries as well as the dissemination of evidence exclusively to decision-makers who had initially expressed an interest in it. The combination of building motivation and opportunity to use evidence succeeded in encouraging decision-makers’ use of evidence as measured by the number of actual evidence-based strategies, policies, and interventions being implemented as well as the reported use of systematic reviews to inform a policy decision in a two-year period.  online database of systematic reviews + weekly targeted messages + KB (knowledge broker) that did not identify a positive effect of applying M3 (communication & access)  - The dissemination of evidence with the engagement of the intended evidence users in the packaging of the evidence. The intervention succeeded in building all three components of behavior change and evidence users reported an increased value and understanding of as well as practical access to the evidence | **Facilitators:**  - Participants who had the option to choose their preferred methods of dissemination (motivation) and accessed the dissemination materials at their own convenience (opportunity and motivation) were also found to be more knowledgeable regarding where to locate relevant evidence materials in general (capability)  - Making systematic reviews more user-friendly by, for example, adding a summary of findings table and plain language summaries.  - Being formally involved in the packaging of the evidence product  - On demand services  - Online repositories  - Apps: creating more convenient and personalized access options and tools.  - Online repositories  - Apps: creating more convenient and personalized access options and tools. | The findings of this project may be of benefit to decision-makers at a practice or policy level who are aiming to make greater use of evidence, and researchers planning to engage in future studies related to EIDM. For decision-makers, this review could hold practical insights on how to enhance the receptivity of their organizational decision-making processes and structures to the use of evidence. They might also benefit from insights on building a professional identity of evidence use with common practices and standards of conduct. Findings related to the reduction of decision making biases and behavioral traps might also be relevant to this audience. Senior decision-makers should consider looking at the role of organizational incentives and protocols to support their staff’s use of evidence | The systematic review of reviews on the impact of evidence use interventions did not include primary evidence and was limited to the data reported in the reviews.  - Included reviews did not always differentiate clearly between interventions and outcomes related to EIDM and interventions and outcomes related to the implementation of evidence based practices; and we could therefore not draw from the full data set reported in some reviews.  - The applied narrative synthesis does not allow us to implement a standardized and comparable effect size measure. It is therefore challenging to establish relative intervention effects and strengths of effects.  - The social science literature was only scoped and we cannot provide an exhaustive account of interventions.  - The identified bodies of social science evidence were often too extensive and featured multiple reviews of different methods and conclusions. For some areas, for example management literature, we could not identify a consensus on what might be the most effective approach relevant to EIDM.  - Some of the suggested social science interventions (and related concepts) might have been tried and applied in EIDM, but have only been reported in primary or theory papers, which were not covered by the systematic review of reviews. We therefore conducted a brief search for primary evidence at the end of the project in key journals such as Evidence & Policy and Implementation Science. | The knowledge gap therefore is to identify which motivational techniques seem to match most effectively with what type of opportunities. There is currently insufficient evidence to comment on the potential of interventions applying M3 (communication & access) to change capabilities to use evidence. While there are individual interventions in which capabilities have increased, by and large, communication & access educational potential appears to be limited. |
| Lavis et al. 2003 [28] | We provide an organizing framework for a knowledge-transfer strategy and an overview of our understanding of the current knowledge for each of the five elements of the framework. | **Information packaging and synthesis tools:** Brief summaries; full summaries | **Electronic tools for dissemination and communication:** Websites  Tools for automated electronic dissemination of information: Newsletters; emails  **Tools for person-to-person communication of health information:**  Meetings  **Information linkage and exchange tools:**  opinion leaders; authoritative endorsement by a respected organization; knowledge brokers; two-way processes (interaction in the research and the decision-making process); one-way (and sometimes one-off) processes (i.e., beyond producer-push efforts) | **Barriers:**  - Not all research can or should have an impact. Some bodies of research knowledge will not generate a “take-home” message, because either the research has no apparent application for decision makers or the findings are not conclusive.  - Accountability mechanisms must be in place to ensure that when take-home messages can be generated, they are appropriate to the decision-making environments to which they are directed  **Perspectives:**  - Research funders can help research organizations take advantage of these opportunities.  - Opportunities for improvement in the research base from which answers to the five questions have been drawn can be found by conducting systematic reviews for each combination of question, target audience, and disciplinary perspective and/or methodological approach. We hope that asking the salient questions, placing them in a logical order, and providing preliminary answers to them will spur such reviews, as well as evaluations of the overall strategy. | Five questions—What should be transferred to decision makers? To whom should research knowledge be transferred? By whom? How? With what effect?—provide an organizing framework for a knowledge-transfer strategy. Opportunities for research organizations’ improvement can be found in the differences between the answers suggested by our understanding of the research literature and those provided by research-organization directors asked to describe what they do. | Not reported | Not reported |
| McCormack et al., 2013 [21] | This systematic review has three related components; all focus on promoting informed decisions about health-related behaviors and decisions among patients and clinicians. First, it addresses the comparative effectiveness of communicating evidence in various contents and formats that increase the likelihood that target audiences will both understand and use the information. Second, it examines the comparative effectiveness of a variety of approaches for disseminating evidence from those who develop it to those who are expected to use it. Third, it examines the comparative effectiveness of various ways of communicating uncertainty associated with health-related evidence to different target audiences, including evidence translators, health educators, patients, and clinicians. | **Electronic tools for dissemination:** The active and targeted distribution of information and interventions to a specific public health or clinical practice audience via determined channels using planned strategies. The intent is to spread knowledge and the associated evidence-based interventions in order to enhance the adoption and the implementation of the information and/or intervention)  • Increase reach to a variety of audiences—Distributing evidence widely to many audiences and across many settings (e.g., postal and electronic mail; electronic/digital, social, and mass media) to increase the reach of information  - Passive dissemination strategies are not as effective as active strategies. Interventions that incorporate two or more distinct strategies (i.e., that are multifaceted) are consistently more likely to work than single interventions.  - Improve reach of evidence: Distributing evidence widely to many audiences and across many settings increases the reach of information  - Postal: Any information delivered via a human carrier employed by a government-run postal service to a new destination or a for-profit mail delivery service  - Electronic and digital media: Any information delivered via phone and/or Web-based e-mail, text messages, or electronic programs such as PDA (personal digital assistant) resources or phone apps  - Social media: Any information delivered via Internet-based social networking sites such as Facebook, Twitter, YouTube, My Space, Foursquare™, LinkedIn, et cetera. Sometimes there are problem or group-specific social networks for professional organizations and patient subgroups; these would also fall into social media as long as they have a “social” network component as described above.  - Mass media: Any information delivered via TV, radio, print newspapers, print magazines, or billboards.  - Interpersonal verbal group or individual outreach: Information delivery via phone, Webinar, or in-person visits, including purposeful delivery of brochures/pamphlets; can include pharmacists, nurses, doctors, counselors, but does not include a motivational component.  - Enhance the ability to use and apply evidence (regardless of delivery mode): Providing additional resources about the evidence, such as how it can be incorporated into current practice or specific suggestions for change, enhances a traditional dissemination strategy  - Provision of supporting “how-to” materials, including physical materials that might be used by a practice to put evidence into use. This might include tracking sheets to be given to patients and risk calculators to be used by clinicians. It might also include tailored toolkits that explain implementation of evidence in specific settings.  - Supporting materials do not include brochures, counseling resources, or resources that originate from the practice. They must originate from the evidence developer and be given to the end-user.  - Skill training, capacity building, and problem solving including training in any skill that would allow appropriate use of evidence (to overcome barriers); might include training in recognizing the quality of evidence or the circumstances under which it can be reasonably used; and also includes training in various counseling techniques that would facilitate evidence implementation and interactive seminars.  - One or more of the above goals/strategies: Combining multiple dissemination strategies—including ways to increase reach, motivation, or ability—may be more effective than single strategies  - A multicomponent approach uses several dissemination strategies in concurrent combination or in sequence to increase the reach of evidence, enhance the end-user(s)’ motivation to use and apply evidence or to adopt it.  Disseminating Evidence to Clinicians  - Ability strategies are not more effective than reach strategies related to clinician behavior (4 trials; low SOE).  - Multicomponent strategies that address a combination of reach, ability, or motivation appear to be more effective than one strategy alone for affecting clinician behaviors, particularly guideline adherence (7 trials; moderate SOE) and for clinical outcomes, although many comparisons examining clinical outcomes were not significant (6 trials; low SOE).  - The SOE is low or insufficient for most comparisons related to clinical outcomes and knowledge for clinicians because we had only single trials in each case.  Disseminating Evidence to Patients  - Evidence is inconsistent for determining the benefit of reach, ability, motivation, or multicomponent approaches for patients focused on changing health-related decisions and behaviors (12 trials; insufficient SOE).  - Evidence is insufficient for determining the benefit of reach, ability, motivation, or multicomponent approaches for patients focused on changing clinical outcomes (2 trials; 1 low SOE, 1 insufficient SOE due to 1 trial in each category).  - Evidence is insufficient for determining the benefit of reach, ability, motivation, or multicomponent approaches for patients focused on changing knowledge outcomes (3 trials; insufficient SOE due to inconsistent findings or 1 trial in a category).  Disseminating Evidence to Patients and Clinicians  - Evidence is inconsistent for determining the benefit of reach, ability, motivation, or multicomponent strategies that target both providers and patients for health-related decisions and behaviors (6 trials; insufficient SOE).  - Evidence is inconsistent for determining the benefit of reach, ability, motivation, or multicomponent strategies that target both providers and patients for health-related decisions and behaviors or clinical outcomes (1 trial in each category; insufficient SOE). | **Tools for person-to-person communication of health information:** Providing messages to participants about their psychological or behavioral states. Individualized feedback may have then been provided synchronously (e.g., via chat, telephone, or face to face) | **Facilitators:**  - Increase motivation to use and apply such information—Increasing interest in the evidence through champions (also known as “cheerleaders”), opinion/thought leaders, or social networks  - Increase ability actually to use and apply evidence—Providing additional resources about the evidence, such as how it can be incorporated into current practice or specific suggestions for change, to enhance a traditional dissemination strategy (e.g., providing additional resources or information; skills-building efforts) | Across the KQs, many of the comparisons yielded insufficient evidence to draw firm conclusions. For KQ 1, we found that investigators frequently blend more than one communication strategy in interventions. For KQ 2, we found that, compared with single dissemination strategies, multicomponent dissemination strategies are more effective at enhancing clinician behavior, particularly for guideline adherence. Key findings for KQ 3 indicate that evidence on communicating overall strength of recommendation and precision was insufficient, but certain ways of communicating directness and net benefit may be helpful in reducing uncertainty. | Limitations for KQ 1 trials included the following:  - The evidence base for addressing comparisons of communication strategies of interest was extremely sparse (i.e., only 7 trials of direct comparisons).  - Trials focused disproportionately on screening interventions. In particular, many trials focused on screening for breast cancer, for which the evidence basis has changed in the recent past. As new evidence emerges in the media, the result can be confusion among patients and the new evidence may produce interference with the impact of interventions.  - Several trials used convenience samples, so unmeasured confounding may exist because of selection bias with the sample.  - All trials used self-reported data, which can be subject to social desirability bias.  Limitations for KQ 2 trials included the following:  - Trials often confounded the mode of distribution with other variables. Therefore, we could not tease apart the effect of mode, channel, and other variables on the outcome of interest.  - Many studies did not consistently compare strategies directly with each other, but instead compared with a usual-care or control condition, or at times made direct comparisons for only some outcomes. This limited our ability to draw conclusions about the comparative effectiveness of one approach versus another.  - The included studies were very heterogeneous with regard to the behaviors, outcomes, targeted populations, and dissemination strategies used. The resulting heterogeneity reflects a commonly encountered attribute of dissemination research. To address this heterogeneous and complicated body of work, we classified the trials in broad terms. Nonetheless, this effort still left too few studies in some categories for making meaningful conclusions about the relative impact of a particular dissemination strategy.  Limitations for KQ 3 trials included the following:  - Trials did not directly test alternative ways to communicate the uncertainty concepts that are relevant to evidence about health and health care. Few studies addressed any type of uncertainty of interest, and none examined ways to communicate risk of bias, consistency across studies, or applicability.  - When acceptable studies were present, we determined that they manipulated relatively limited comparisons. For instance, few alternative wordings were tested for communicating strength of evidence, and few graphical presentations were tested for communicating precision.  - Few studies were directed toward clinicians. | Major gaps across the KQs include (1) testing communication strategies (e.g., targeting, tailoring, or narratives) with clinicians; (2) testing dissemination strategies that are not confounded by mode of delivery, are informed by the target audience’s needs, and are supported by theory; (3) testing communication studies that address uncertainty for clinicians or examine communicating risk of bias, consistency, or applicability of the evidence.  Research teams should try to address not only the conceptual and study limitations noted for each KQ, above, but also the methodological recommendations noted below:  - Relying more on accepted theoretical constructs and models when designing interventions and studies  - Conducting some prior-needs assessments with target audiences, focusing on audience subgroups with greatest needs  - Designing robust trials or observational studies  - Using an array of proven data collection methods that can include, but might go beyond, self-reported attitudes, levels of knowledge, and behaviors  - Describing and defending choices of intermediate and ultimate outcomes  - Applying modeling or other advanced statistical and analytic techniques to account for confounders, interactions, and similar complications in data, and addressing temporal aspects of outcomes  - Thoroughly describing all aspects of study design and conduct, especially for interventions  The lack of comparative research evidence to inform communication and dissemination of evidence, including uncertain evidence, impedes timely clinician, patient, and policymaker awareness, uptake, and use of evidence to improve the quality of care. Expanding investment in communication, dissemination, and implementation research is critical to the identification of strategies to accelerate the translation of comparative effectiveness research into community and clinical practice and the direct benefit of patient care. |
| Meisel et al. 2019 [22] | To identify barriers and facilitators, attitudes, beliefs, and experiences surrounding the use of research related to the treatment and economics of substance use disorder. | Not reported | Not reported | **Needs:**  - To differentiate between generalizable information and local data;  - Researchers to be directly engaged in policy-making dialogues.  **Facilitators:**  - Engagement with evidence and evidence producers: Personal and professional relationships are key components of policy makers’ engagement.  **Barriers:**  - Policy stakeholders spoke extensively about their frustrations with research that suffered from jargon, overly technical methodological approaches, poor timing (slow speed from idea to publication), and research designs or data sources that didn’t feel relevant to their needs.  **Perspectives:**  - Systematically test alternative phrasing of scientific terminology – particularly in the realm of cost effectiveness research – that allow end users to better understand and repurpose the data;  - Promote and reward researcher involvement in policy discussions. | In summary, policymakers want research and researchers to help them understand and be able to convey the importance and feasibility of various SUD policy solutions. | Although we identified themes common to participants, there may be limits to generalizability because of the snowball sampling strategy employed. Participants may have been susceptible to social desirability bias in which they reported favorable perspectives on research because they were speaking to researchers. Although infectious disease (HIV and Hepatitis C) screening and treatment was a pre-specified topic for investigation, the analysis did not uncover specific ideas or concepts related to this topic that were distinct from the overall themes which emerged during the general discussion of substance use and economics evidence. Finally, an important limitation is that awareness, use, and attitudes toward evidence-based policies in substance use disorder are likely evolve over time. | Perceptions may change with publication of new evidence, media attention, passage of legislation, dissemination of resources, evolution of the current opioid crisis, or new substance use disorder related public health challenges. |
| Oliver et al. 2014 [8] | This review aimed to update and expand Innvaer, and broaden the scope of the review to:  - Identify factors which act as barriers to and facilitators of the use of evidence in public policy, including factors perceived by different stakeholder groups;  - Describe the focus, methods, populations, and findings of the new evidence in this area. | Not reported | Not reported | **Facilitators:**  - Availability and access to research/ improved dissemination  - Collaboration  - Clarity/ relevance/ reliability of research findings  - Relationship with policymakers  - Relationship with researchers/info staff  - Incentives to use evidence and client demand for research evidence were described as facilitators  **Barriers:**  - Availability and access to research/ improved dissemination  - Clarity/ relevance/ reliability of research findings  - Timing/ opportunity  - Policymaker research skills | This review looked for all barriers and facilitators of the used of evidence in policy. Most studies collected research and policy actors’ perceptions about factors affecting the use of research evidence, with a large minority surveying only researchers. Understanding how to alleviate these barriers is hampered by a lack of clarity about how evidence’ is defined by studies, with fewer than half specifying what kinds of information were discussed. Most studies however focused on uptake of research evidence, as opposed to evidence more widely. Research into how to alleviate organizational and resource barriers effectively would be welcomed. Additionally, all such research should be based on an understanding that a broader interpretation of “evidence” than “research-based” evidence is also essential. | Most studies still employ relatively superficial methods such as surveys or short interviews. These were all based on self-reports, however, so given the contentious nature of the topic combined with understandable fear of audit/performance monitoring these results may not be reliable. We were unable to double-screen and double-code all studies due to lack of resources. No methodological assessment of included studies was undertaken, as this was primarily a descriptive exercise. In addition, the heterogeneity of study designs and the difficulty of comparing quality across these domains limited the usefulness of such an exercise. Quality appraisal would be a valuable step in any in-depth review of a subset of these studies. | The reviews all found similar findings with regard to barriers and facilitators of the use of evidence. There still appears to be a need for high-quality, simple, clear and relevant research summaries, to be delivered by known and trusted researchers. |
| Purtle et al. 2020 [23] | To synthesize the results of empirical studies to provide guidance about how to enhance the dissemination of children’s mental health services research to policy makers. | **Information packaging and synthesis tools:**  Research brief; evidence summaries; policy briefs; economic evaluation data  **Visualization and modelling tools:**  Maps using geographic information systems, policy map (a web-based data mapping tool); strategic frames (for example, brief narratives or stories) | **Tools for person-to-person communication of health information:**  Seminars | **Needs:**  - Researchers might consider adding details about the study design, p values, and confidence intervals — while also keeping the information presented concise.  **Facilitators:**  Curate content of dissemination materials  - Include economic evaluation data, such as information about the cost-effectiveness of various children’s mental health services and budget impacts of investments in children’s mental health service systems.  -Use state and local data that correspond with the jurisdiction of the policy maker when presenting evidence about the prevalence of children’s mental health issues and service availability.  -Keep dissemination materials concise and to the point. Policy makers value brevity.  -Emphasize evidence in dissemination materials that target legislators who prioritize behavioral health issues (e.g. legislators who introduce behavioral health bills).  - Use strategic frames and tailor materials for different audiences  - Use stories to illustrate how systems and structural factors influence children’s mental health outcomes.  - Emphasize that factors beyond the control of children and their families affect mental health outcomes.  -Be sensitive to the fact that stigma toward children with mental illness is pervasive among state legislators and their constituents.  - Recognize that framing investments in children’ mental health as a strategy to prevent mass shootings is likely to produce and perpetuate stigma.  - Recognize that Democrat and Republican (and liberal and conservative) legislators have different behavioral health dissemination preferences, knowledge about children’s mental health issues, and opinions about the effectiveness of mental health services.  - Preparing and presenting maps using geographic information systems is a potentially effective way to demonstrate the local relevance of an issue to policy makers  - Use intermediary organizations to ensure reach of dissemination materials  -Account for the fact that elected and administrative policy makers turn to different sources for behavioral health evidence—with mental health advocacy organizations being the primary sources for legislators and professional organizations being the primary source for state mental health agency officials.  - Studies suggest that details about empirical evidence should be explicitly emphasized, not glossed over, when communicating with legislators who prioritize behavioral health issues.  - Message tailoring and audience segmentation.  **Barriers:**  - lack of time  - lack of clear summary of research findings  - presenting too much evidence can be counterproductive.  - increasing the amount of information in an evidence summary did not produce beliefs that were aligned with the evidence and in some cases amplified beliefs that were counter to the evidence. | Like most issues in the realm of children’s mental health, the problem that policy maker–focused dissemination seeks to solve is extremely complex. It would be naive to think that dissemination strategies, even if designed and executed with absolute precision, would transform the policy environment. That said, the effectiveness of dissemination strategies can certainly be enhanced. Specifically, policy maker–focused dissemination strategies can be improved by using empirical data to inform decisions about what information is included in dissemination materials, how evidence is framed for different audiences, and the entities that deliver dissemination materials. Dissemination science can generate these data and help accelerate the policy impact of children’s mental health services research. | The data presented in this article about the dissemination preferences of administrative policy makers are limited to those in state mental health agencies and policy makers across a range of executive branch agencies who make decisions that affect children’s mental health. | Although some prior work has been conducted in this area, there could be benefit to future research with a specific eye toward implications for dissemination. Finally, there is a need for experimental research that tests the effects of different dissemination strategies on policy makers’ engagement with and uses of children’s mental health research, knowledge and attitudes about children’s mental health issues, and policy-making behaviors (e.g., volume and content of children’s mental health policy proposals). |
| Schmidt et al. 2014 [24] | To understand the motivations and priorities of policymakers in North Carolina, a state that enacted a strong tobacco control program from 2003–2011, but drastically reduced funding in recent years. | **Information packaging and synthesis tools:**  Policy briefs were presented, interviewees emphasized the importance of succinct  information | Not reported | **Needs:**  - Concise, Contextualized Messages  **Facilitators:**  - Personal contact: Messages were most effective when delivered in person by individuals directly impacted by tobacco, especially survivors of tobacco use from a legislator’s home district.  **Barriers:**  - Limited time.  **Perspectives:**  - Interviewees indicated that merely presenting facts and statistics was insufficient. Several participants mentioned wanting numerical data to be put into context. They felt this would help them to digest the statistics and appreciate and understand their relevance. | These findings suggest that building relationships with policymakers to communicate ongoing program outcomes, emphasizing economic data, and developing a constituent advocacy group would be valuable to encourage continued support of state tobacco control programs. | Sample size was small, state funding in the interview guide was conceptualized as MSA monies, which is not the only source of funds available to states for tobacco control. Last, as North Carolina is a top tobacco producing state, its legislators may be somewhat more hesitant to support tobacco control than the average state. | As a next step, practitioners and researchers should more widely test the recommendations and those found in the literature to identify best practices and successfully securing funding for critical state tobacco control programs. |
| Wye et al. 2015 [26] | To elucidate the reasons that prompted commissioners to seek information, to clarify which sources and types of knowledge commissioners commonly consulted and to describe the use of research evidence in decision-making. | **Information packaging and synthesis tools:**  Evidence summaries;  meeting papers; NICE guidance and guidelines; guidelines, pathways | **Electronic tools for dissemination and communication: G**oogle and Google Scholar, apart from the British Medical Journal and the British Journal of General Practice.  **Tools for person-to-person communication of health information:** chance encounters, formal meetings and informal gatherings | **Barriers:** about use of academic research  - The challenges of finding applicable, relevant research for commissioning, given that most health related academic research was clinically focused;  - “Academically very robust” interventions not working in practice  - The challenge of drawing conclusions from literature reviews with substantial variations in interventions;  - Lack of commissioner time and skills to do comprehensive reviews;  - Difficulties in interpreting the “spin” within abstracts;  - The length of time needed for locally commissioned research to produce meaningful outputs;  - Challenges in applying negative or inconclusive findings, as commissioners’ focus was on identifying initiatives that might work.  **Perspectives:**  - This suggests that the ‘problem’ is less about commissioners having to change their information acquisition habits and more about the way that academics generate research;  - Academic researchers need to engage with commissioners using commissioners’ preferred methods of conversations and stories, to find out what is wanted and how best to deliver it;  - Researchers need to learn more about local commissioning priorities to produce more relevant, useful research;  - Researchers need to learn to package their messages in commissioner-friendly ways;  - Researchers can build relationships with intermediaries with their local public health departments whose staff understand the value of research and have wide commissioner networks; | By juggling competing agendas, priorities, power relationships, demands and personal inclinations, commissioners built persuasive, compelling arguments to inform local commissioning decisions. They sought information to identify options, navigate a way through the system, justify decisions and convince internal and external parties to approve and/or follow the suggested course. | - With documentary data, we were also sometimes able to track information as it moved through the system. However, we were not in the field constantly. Sometimes the information disappeared, morphed or reappeared elsewhere, without our knowledge.  - A further limitation in common with other ethnographic studies is that we do not know to what extent the presence of researchers changed the dynamics of the meetings observed. | Not reported |

**Acronyms:** HEN - health evidence network; HI - health information; ICT - information and communication technology; KT - knowledge translation; MSA -master settlement agreement ; US - United States.

Appendix 4.1. Methodological assessment, using the JBI Critical Appraisal Checklist for systematic reviews and research syntheses

| Study (in alphabetic order) | 1. Is the review question clearly and explicitly stated? | 2. Were the inclusion criteria appropriate for the review question? | 3. Was the search strategy appropriate? | 4. Were the sources and resources used to search for studies adequate? | 5. Were the criteria for appraising studies appropriate? | 6. Was critical appraisal conducted by two or more reviewers independently? | 7. Were there methods to minimize errors in data extraction? | 8. Were the methods used to combine studies appropriate? | 9. Was the likelihood of publication bias assessed? | 10. Were recommendations for policy and/or practice supported by the reported data? | 11. Were the specific directives for new research appropriate? |
| --- | --- | --- | --- | --- | --- | --- | --- | --- | --- | --- | --- |
| Ashcraft et al. 2020 [29] | Yes | Yes | Yes | Yes | No | No | Yes | Yes | No | Yes | Yes |
| Blessing et al. 2017 [18] | Yes | Yes | Yes | Yes | No | Not applicable | No | Yes | Not applicable | Yes | Yes |
| Chapman et al. 2021 [12] | Yes | Yes | Yes | Yes | Yes | Yes | Yes | Yes | Not applicable | Yes | Yes |
| Funk et al., 2021 [19] | Yes | Yes | Yes | Yes | Not applicable | Not applicable | No | Yes | Not applicable | Yes | Yes |
| Innvaer et al. 2002 [27] | Yes | Yes | Yes | Yes | No | Not applicable | No | Yes | Not applicable | Yes | Yes |
| Langer et al. 2016 [20] | Yes | Yes | Yes | Yes | Yes | No | Yes | Yes | No | Yes | Yes |
| McCormack et al. 2013 [21] | Yes | Yes | Yes | Yes | Yes | No | Yes | Yes | No | Yes | Yes |
| Oliver et al., 2014 [8] | Yes | Yes | Yes | Yes | No | No | Yes | Yes | Not applicable | Yes | Yes |

Appendix 4.2. Methodological assessment, using the JBI Critical appraisal checklist for qualitative research

| Study (in alphabetic order) | 1. Is there congruity between the stated philosophical perspective and the research methodology? | 2. Is there congruity between the research methodology and the research question or objectives? | 3. Is there congruity between the research methodology and the methods used to collect data? | 4. Is there congruity between the research methodology and the representation and analysis of data? | 5. Is there congruity between the research methodology and the interpretation of results? | 6. Is there a statement locating the researcher culturally or theoretically? | 7. Is the influence of the researcher on the research, and vice- versa, addressed? | 8. Are participants, and their voices, adequately represented? | 9. Is the research ethical according to current criteria or, for recent studies, and is there evidence of ethical approval by an appropriate body? | 10. Do the conclusions drawn in the research report flow from the analysis, or interpretation, of the data? |
| --- | --- | --- | --- | --- | --- | --- | --- | --- | --- | --- |
| Campbell et al. 2009 [30] | Yes | Yes | Yes | Yes | Yes | No | No | Yes | No | Yes |
| Dobbins et al. 2004 [25] | Yes | Yes | Yes | Yes | Yes | Yes | Yes | Yes | Yes | Yes |
| Dobbins et al., 2007 [5] | Yes | Yes | Yes | Yes | Yes | Yes | No | Yes | Yes | Yes |
| Lavis et al., 2003 [28] | Yes | Yes | Yes | Yes | Yes | Yes | No | Yes | No | Yes |
| Meisel et al. 2019 [22] | Yes | Yes | Yes | Yes | Yes | No | Yes | Yes | Yes | Yes |
| Schmidt et al. 2014 [24] | Yes | Yes | Yes | Yes | Yes | No | No | Yes | Yes | Yes |
| Wye et al., 2015 [26] | Yes | Yes | Yes | Yes | Yes | Yes | No | Yes | Yes | Yes |

Appendix 4.3. Methodological assessment, using the Scale for the Assessment of Narrative Review Articles (SANRA)

| Study | 1.Justification of the article's importance for the readership | 2.Statement of concrete aims or formulation of questions | 3. Description of the literature search | 4. Referencing | 5. Scientific reasoning | 6. Appropriate presentation of data | Total |
| --- | --- | --- | --- | --- | --- | --- | --- |
| Purtle et al, 2020 [23] | 2- The importance is explicitly justified | 2-One or more concrete aims or questions are formulated | 0-The search strategy is not presented | 2-Key statements are supported by references | 0-The article's point is not based on appropriate arguments | 2- Relevant outcome data are generally presented appropriately | 8/12 |

Appendix 5. Strategies for evidence communication and dissemination (Blessing et al. [18]; Funk et al. [19]; Langer et al. [20]; McCormarck et al. [21])

| **Strategies for evidence communication** | |
| --- | --- |
| **Strategy** | **Explanation** |
| Targeting the message | - Manipulating language, visuals, music, or choice of behavior topic in ways that make the message more interesting, relevant, or appealing to specific subgroups [21]. |
|  |  |
| Tailoring the message | - Using a computerized database of messages that can be combined in response to answers to preprogrammed questions asked of an individual [21]. - Applying an electronic algorithm to design messages based on individual input regarding a limited number of questions [21]. - Trying to direct messages to individuals’ status on key theoretical determinants (knowledge, outcome expectations, normative beliefs, efficacy. or skills) of the behavior of interest [21]. - Incorporating recognizable aspects of participants to convey (implicitly or explicitly) that the messages are designed specifically for them. This is more than a personalized letter (e.g., “Dear Jane”) [21]. - Providing messages to participants about their psychological or behavioral states. Individualized feedback may be provided synchronously (e.g., via a chat function, telephone, or face-to-face) or asynchronously (e.g., via email or discussion board, or mail) [21]. - Using messages that are personalized based on an individual’s unique psychological characteristics (e.g., ambiguity aversion, optimism) that might affect their interpretation of evidence [21]. |
| Tailoring & targeting | - Potential to align the communication of evidence to decision-makers’ professional needs and personal preference to increase motivation to use evidence (strong evidence) [20]. |
| Information design | - Potencial para aumentar a acessibilidade, bem como o apelo visual da evidência para aumentar a motivação para usar a evidência (weak evidence) [20]. |
| Health information packaging tools | - Synthesis tools: Format that brings together existing evidence, involving the aggregation of explicit and tacit knowledge and with information presented in a way that can be readily used by stakeholders [18]. Visualization tools: For health information to be useful, it needs to be analyzed and communicated in a way that is easy to use and practical, allowing both specialists and non-specialists to understand and use it. Presenting health information visually can help users to capture and comprehend complex information more quickly, and, therefore, makes it easier to draw conclusions [18]. |
| Application tools | - Modelling and simulation tools: A model is a simplified, mathematical representation of a real phenomenon, often built on existing data and variable parameters. Simulation tools can, in turn, extend such models by applying an algorithm and exploring the behavior and performance of processes and interventions [18]. - Integrated public health surveillance platforms: Public health surveillance programs are part of national public health systems and often provide a platform for ongoing systematic collection, analysis and interpretation of health data, coupled with timely dissemination to support public health action [18]. |
| Using narratives | - Invoking personal stories, case studies, anecdotes, testimonials, experiential sharing (e.g., personal account of an individual’s experience in donating an organ to a sibling) [21]. - Using entertainment education (e.g., talking about issue in a soap opera storyline) or photo novellas or graphic novels [21]. - Potential to increase the relevance and accessibility of research evidence or the concept of EIDM through emotional connections and identification (strong evidence) [20]. |
| Framing the message (gain/loss) | - Creating messages that emphasize the positive consequences of compliance are referred to as positive (gain) frame, whereas those that stress the negative consequence of noncompliance are denoted as negative (loss) frame. Studies should explicitly state that the stimuli differed in terms of gain or loss frame. For example,   Positive (gain) frame: “Get active! Enhance your health!” vs. “A lack of activity increases risk for diabetes.”  Negative (loss) frame: “With drug X, you have a 5% chance of dying” vs. “With drug X, you have a 95% chance of surviving” [21].   - Potential to align the communication of the results of research with the cognitive characteristics of the decision or the desired behavior (i.e. evidence use). Framing increases the likelihood that information will be considered and taken into account potentially affecting opportunity and motivation to use evidence (strong evidence) [20]. |
| Framing (norms / identities); identify cues & priming | - Potential to align the communication of evidence or the concept of EIDM with the decision-makers’ existing norms and identity. Identity cues and frames can increase motivation to use evidence (weak evidence) [20]. |
| Explaining uncertainty | - Potential to use communication techniques to explain uncertainty in the results of research to increase decision-makers’ engagement with research (i.e. motivation) (strong evidence) [20]. - Non-numeric presentations: Using words or sentences to describe the presence, degree, or meaning of uncertainty in medical evidence [21]. - Numeric presentations: Using numbers to describe the presence, degree, or meaning of uncertainty in medical evidence [21]. - Using graphs, images, or figures to describe the presence, degree, or meaning of uncertainty in medical evidence [21]. - Manipulating the presentation of uncertainty to make it more interesting, relevant, or appealing to a specific subgroup of individuals [21]. - Invoking personal stories, case studies, anecdotes, or testimonials to help individuals understand the presence, degree, or meaning of uncertainty related to medical evidence [21]. - Creating messages that present uncertainty in alternate contexts (e.g., relative to other more or less uncertain services) [21]. - Creating messages that present alternate consequences of uncertainty (e.g., “chances may be as high as” or “chances may be as low as”) [21]. |
| More than one of the above strategies | - A multicomponent approach uses several communication strategies in concurrent combination or in sequence to increase understanding of the evidence or information [21]. - Multicomponent interventions are important to this review only to the extent that they are compared with another intervention that is different by only 1 or more aspects [21]. |
| **Strategies for evidence dissemination** | |
| **Strategy** | **Explanation** |
| Audience segmentation | - Potential to ensure that the communicated evidence is adapted to each target audience to increase reception and motivation. (unclear, no independent evidence) [20]. |
| Improve reach of evidence | - Postal: Any information delivered to a new destination via a human carrier employed by a government-affiliated postal service or a for-profit mail or parcel delivery service such as FedEx™ or UPS® [21]. |
|  | - Electronic and digital media: Any information delivered via telephone or web-based email, text messages, or electronic programs such as personal digital assistant (PDA) resources or phone apps [21]. - Apps: Potential to create more convenient access options and tools on mobile devices. Evidence use apps might not present the standard type of access but might be able to provide an appealing and personalized first encounter with EIDM (motivation and opportunity) (No evidence identified) [20]. |
|  | - Social media: Any information delivered via Internet-based social networking sites such as Facebook, Twitter, YouTube, Myspace, foursquare, and LinkedIn. Sometimes problem- or group-specific social networks exist for professional organizations or patient subgroups; these would fall into social media as long as they have a “social” network component as described above [21]. |
|  | - Mass media: Any information delivered via television, radio, print newspapers, print magazines, or billboards [21]. |
|  | - Interpersonal verbal group or individual outreach: Information delivered via telephone, webinar, or in-person visits, including purposeful delivery of brochures or pamphlets, but without any motivational component. The audiences can include: pharmacists, nurses, doctors, counselors, or other clinicians [21]. |
|  | - Reminders: Potential to reinforce communicated research results, triggered frames, and targeted behavior of accessing evidence. Reminders can affect instant motivation but also provide an opportunity to reinforce intended accessing of evidence (strong evidence) [20]. |
| Online and social media | - Potential to increase the reach and convenience of access to evidence communication or the concept of EIDM (i.e. motivation and opportunity) (strong evidence) [20]. |
| Dissemination and communication tools | - Electronic tools for dissemination and communication: Platforms for sharing health information allow users to select and access health information electronically. Some provide information to users, others let users link up with each other in networks to share information, and some fulfil both of these functions [18]. - Online repositories: Potential to create more effective online platforms if IT-design principles are emphasized in addition to functionality. This might increase user’s motivation to use the repositories and thus increase opportunity to use evidence. (No evidence identified) [20]. - Tools for automated electronic dissemination of information: Health information can be electronically delivered to stakeholders directly. Newsletters, email messages, tweets or phone messages are all tools that can be used to disseminate information in this way [18]. - Tools for person-to-person communication of health information: Health information can also be disseminated through personal contacts. By exchanging knowledge on a personal level, trust can be created between stakeholders, which, in turn, increases the likelihood of health information being [18]. |
| Branding | - Potential to increase the credibility, visibility, and emotional connection of the concept of EIDM. Branding could support motivation to use but also affect behavioral intentions and change (strong evidence) [20]. |
| Timing | - Potential to increase the timing of evidence communication to receptive periods of decision-makers / the decision-making process (i.e. opportunity) (weak evidence) [20]. |
| Linkage and exchange tools | - Tools used to achieve linkage and exchange are dedicated to groups of stakeholders, knowledge networks and individual knowledge brokers; they are used to facilitate exchange and institutionalization of knowledge translation. Stakeholder groups are brought together within dedicated structures or semi-permanent groups with a defined membership and regular meetings [18]. |
| Motivate recipients to use and apply evidence | - Champions (cheerleaders): People who take ownership of the evidence and visibly promotes it within their own organization or across other settings. Champions help overcome social and political pressures imposed by an organization, provide a role model for personal commitment to the program, and involve others in its use [21]. For example, an evidence developer might train or enlist the help of a local champion to promote evidence within his or her organization. - Opinion or thought leaders (frequently has an endorsing or persuasive element): Recognized experts who lend their name to dissemination efforts to endorse the idea being disseminated and to establish credibility. They may or may not actually participate in the work and do not necessarily have any relationship with the organization to which evidence is to be disseminated. They could endorse the intervention, have a role in its development, or advise on strategies [21]. For example, an opinion leader might be the CEO or the head of a department, an external expert in a particular field applicable to the evidence, or a well-recognized figure such as the U.S. Surgeon General. - Social networks: A network of individuals who have a common perspective, relationships, or similar connection. The relationships can be informal (friends, peers, family) or formal (patient provider, nurses), but network members have defined role obligations. Peer networks provide a central and trusted source for information and might use multiple other dissemination strategies themselves (such as newsletters, journals, phone- and internet-based distribution, face-to-face conferences, peer-to-peer conversations, etc.). [21]. |
| Enhance recipients’ ability to use and apply evidence (regardless of delivery mode) | - Provision of supporting “how-to” materials: Includes physical materials that a health care practice might use to apply evidence in their activities. These might include giving tracking sheets to patients or giving risk calculators to clinicians. These might also include tailored toolkits that explain how to implement evidence-based recommendations from in specific settings [21]. - Supporting materials do not include brochures, counseling resources, or resources that originate from the practice. They must originate from the evidence developer and be given to the end user [21]. - Skill training, capacity building, and problem solving: Training in any skill that would allow appropriate use of evidence (to overcome barriers); might include training in recognizing the quality of evidence or the circumstances under which it can be reasonably used; also includes training in various counseling techniques that would facilitate evidence implementation and interactive seminars [21]. |
| More than one of the above strategies | - A multicomponent approach uses several dissemination strategies in concurrent combination or in sequence to increase the reach of evidence, enhance the end users’ motivation to adopt and use or apply evidence. Multicomponent interventions are important to this review only to the extent that they are compared with another intervention that is different by at least one other aspect [21]. |

Appendix 6. Barriers and facilitators based on the toolkit proposed by the Registered Nurses' Association of Ontario [32].

| **Level** | **Barriers** | **Facilitators** |
| --- | --- | --- |
| **Micro (individual) level**  Areas to reflect on include:   - Health providers’ knowledge, attitudes and beliefs - Health providers’ competencies in carrying out the change - Characteristics of patients/persons and families (if applicable) | **Inadequate format for presenting evidence**  In this regard, weaknesses were reported in the presentation of research results[8,12,30] and information overload[23,25] receiving information from different sources with difficulty in categorizing and assimilating this large amount of material[25], excess of jargon and scientific terms[22] and lack of an objective executive summary[23,26]. In some cases, a greater chance of making a decision contrary to the evidence presented was reported [23].  **Difficulty in accessing evidence**  The difficulties were based on locating the evidence, since policy-makers do not seem to have access to it when necessary[8,12,30], and the time pressures for decision-making[30], not being possible, often, that evidence is generated in time to be used[8,12,22,23,24,26,27].  **Lack of relevant or good quality research**  Policy-makers report that research is often inconclusive [28, 29], of low quality [8,27,29] and without relevance to its context [12,22,29,30]. The reasons given for the lack of relevance of research are that present interventions with little applicability to the real world [26].  **Lack of ability to deal with evidence**  Studies indicate that there is a lack of training for policy-makers to be able to read the studies critically [8,12] and to draw their own conclusions[12,26].  **Little collaboration between researchers and policy-makers**  This type of barrier was reported as the lack of dialogue and pre-established partnerships [12,27], the non-involvement of policy-makers in research activities that could favor communication and the use of evidence, such as the participation of policy-makers in the stages of analysis, writing and publication of results[30] and the lack of alignment between the interests of stakeholders[12]. | **Format and content of materials**  The studies reported some aspects of the products used to communicate evidence that are perceived as positive by policy-makers. For example, researchers need to pay attention to the amount of information passed on, taking care that it is well dosed [22,25]. The message needs to be simple, well written, clear, concise, easy to read, transparent and well organized [8,12,27]. The good quality of the materials presented was also another point mentioned [8,27]. For Dobbins and collaborators[ 25], a work is considered of quality when it brings up-to-date data, contextualized at local, regional or national levels, does not use scientific research jargon, and the content is transparent and organized to show what works and what that doesn't work. Policy-makers perceive the inclusion of effectiveness data [27], and cost analyzes[22,25] as positive. Regarding format and content, there is a preference for varied and accessible formats of materials, the presentation of evidence summaries, with clear recommendations [27] and maps with geographic information to contextualize the information[22]. One study pointed that making systematic reviews more user-friendly by, for example, adding a summary of findings table and plain language summaries [20], another showed that providing additional resources about the evidence, such as how it can be incorporated into current practice or specific suggestions for change, to enhance a traditional dissemination strategy [21].  **Relevance of the information produced for policy-makers**  It is an aspect that emerged as a facilitator of communication and dissemination of results in several studies [23,27,25]. Research with credibility and application to the local context was considered relevant.  **Customized and specific products for the target audience**  Studies point to the need to offer personalized products [8,23,29] or services on demand [20]. One study showed that while lawmakers prefer to read printed materials, their advisers are more likely to read documents online. However, this preference seems to be influenced by the age of the policy-maker, with the younger ones seeming to have an easier time reading electronic materials [29]. Another study pointed out that electronic formats were preferred by most policy-makers, but some of them reported that they sometimes miss a physical copy, especially when the document is longer or they need to send it to other people by mail [25]. One study showed that participants who had the option to choose their preferred methods of dissemination (motivation) and accessed the dissemination materials at their own convenience (opportunity and motivation) were found to be more knowledgeable regarding where to locate relevant evidence materials in general (capability) [20].  **Ease of access to information**  In addition to the information being well presented, in different channels, being relevant and designed according to the target audience, for dissemination and communication to occur, it is necessary for policy-makers to be able to access it in a timely manner for use[27,25], with agility [25] and appropriate to the themes worked by them at that moment [25]. Timely access is intrinsically related to the ease of access to information [8,25]. Therefore, the availability of electronic repositories for the materials produced, addresses/links that work correctly [25], online repositories [20], Apps to create more convenient and personalized access options and tools [20] appear as facilitators.  **Relationship between researchers and policy-makers**  Research results indicate that having a pre-established relationship with researchers [8,22,27], and easy access to them when necessary[30] promotes communication, dissemination and use of evidence by policy-makers. This pre-established dialogue increases trust between the parties and policy-makers' understanding of the research results [27]. In addition, researchers with the ability to show the relevance of research to policy-makers, find it easier to engage with these decision makers [29]. |
| **Meso (organizational) level**  Areas to reflect on include:   - Leadership - Culture - Resources available | **Absence of institutionalization/culture for the use of scientific evidence**  Policy-makers do not always seem sensitized to the use of evidence in decision-making [12,29], they are not interested in considering it or are unaware of the recommendations for its use in the formulation of policies[12,30]. Other difficulties reported were the absence of institutionalized channels for communication/dissemination between researchers and policy-makers [12].  **Lack of material/human resources**  The difficulties refer to the lack of access to the internet, printers, librarians, consultants and research assistants to help locate and interpret the information received [25] and lack of financial resources for the production and dissemination of evidence[12,25,27]. The frequent rotation of the teams also appeared to hinder this process [12]. | **Diversity in communication and dissemination channels**  Another beneficial aspect is the use of different channels of communication and dissemination of evidence [23,24]. It is important to use multiple approaches, such intermediary organizations [23]. One of the studies reported that the delivery of information was more effective when delivered to policy-makers by people directly affected by a certain intervention [24].  **Participation of knowledge brokers**  One positive aspect mentioned is increasing interest in the evidence through champions (also known as “cheerleaders”), opinion/thought leaders, or social networks [21].  **Encouraging the use of evidence and knowledge translation plans**  Encouraging the use of evidence by the community [27] and consumers [8] together with the use of knowledge translation plans (n=1). Another important aspect was being formally involved in the packaging of the evidence product [20]. |
| **Macro (system level)**  Areas to reflect on include:   - Whether the policies and governmental standards may become barriers for the change - Whether aspects of the change are in line with existing policies, government standards | **Political/organizational instability**  Among the barriers were political or organizational instability [27]. | Not reported. |

Appendix 7. Future perspectives to improve communication between researchers and policy-makers

| **Level** | **Strategies to overcome barriers** |
| --- | --- |
| **Micro (individual) level**  Areas to reflect on include:   - Health providers’ knowledge, attitudes and beliefs - Health providers’ competencies in carrying out the change - Characteristics of patients/persons and families (if applicable) | **Access to evidence**  Send information at the right time for decision-making [25].  **Format/Content of Materials/ Mode of Delivery**  Adapt the dosage of the information delivered [23,25]; Dedicate more time and effort to writing executive summaries [25]; Deliver material in a timely and familiar format [25]; Differentiate local and global evidence [22]; Highlight implications for practice and policy [5]; Highlight the main lesson [5]; Improve ways of effectively transmitting key information from systematic reviews, in various channels and formats/products [4]; Present preliminary information [28];Produce and deliver executive summary [4]; Produce customized information (format and mode of delivery) [23,25,28]; Provide clear information about what works and what doesn't for different contexts and populations[4];Provide different types of studies and products ("synthesis of local data, brief research summary, systematic reviews") [30]; Provide methodological details, such as p-value and confidence intervals, keeping the information concise[23]; Systematically test new scientific terms to facilitate the understanding of research [22].  **Information relevance**  Addressing aspects of implementation in practice and policy formulation [3,26]; Regularly update research in the policy-makers' area of interest [25]; Promote discussions on relevance, implications for practice and implementation(3); Align researchers with priority problems [25,26].  **Relationship between researchers and policy-makers**  Directly involve researchers in policy-making dialogues [22]; Engage policy-makers permanently in research [26,29,30]; Establish research units in which policy-makers have easy access to researchers and knowledge brokers [30]; Promote and reward researchers' involvement in policy discussions [22); Promote mutual trust [26]. |
| **Meso (organizational) level**  Areas to reflect on include:   - Leadership - Culture - Resources available | **Institutionalization/Culture for the use of scientific evidence**  Encourage funders' support in communicating evidence more adequately [28]; Evaluate the strategies used [28]; Improve alignment between researchers, implementers and policymakers [28]; Strengthen evidence-informed policies [30]. |
